# Features and Differences in Core Symptom Clusters in Home‐Based Hospice Patients With Advanced Cancer: A Network Analysis

**DOI:** 10.1002/cam4.70370

**Published:** 2024-11-04

**Authors:** Yitao Wei, Wan Cheng, Yuanfeng Lu, Zheng Zhu, Guiru Xu, Hong Wu, Shaowei Lin, Huimin Xiao

**Affiliations:** ^1^ School of Nursing Fujian Medical University Fuzhou China; ^2^ School of Nursing Fudan University Shanghai China; ^3^ Department of Hospice Care Fujian Provincial Hospital Fuzhou China; ^4^ School of Public Health Fujian Medical University Fuzhou China; ^5^ Research Center for Nursing Humanity Fujian Medical University Fuzhou China

**Keywords:** advanced cancer, home‐based hospice, network analysis, nutrition impact symptom, symptom cluster, symptom network

## Abstract

**Introduction:**

Patients with terminal‐stage cancer frequently experience multiple symptoms simultaneously. Little is known about how core symptom clusters differ in advanced‐cancer patients with different survival expectancies receiving hospice care. To identify the core symptom clusters of hospice‐care cancer patients with different survival expectancies and compare the features of their symptom networks.

**Methods:**

In this retrospective study, secondary data analysis was conducted. Records of 6946 patients with advanced cancer who received home‐based hospice care service in a hospice center from April 2001 to December 2020 were collected and analyzed using network analysis.

**Results:**

This analysis included 6946 patients with advanced cancer receiving hospice care. In patients with survival expectancies of 0–6 months, loss of appetite was identified as the core symptom (rs = 4.03, rb = 5.21, rc = 2.63), and five symptom clusters were identified. Malnutrition was the core symptom in patients with survival expectancies of 6–12 months (rs = 2.83, rb = 2.43, rc = 0.93), and nine symptom clusters were identified. Wasting syndrome was the core symptom cluster in two groups. The network density of symptoms in patients with < 6 months of survival expectancy (91.99) was higher than in patients with 6–12 months (28.39).

**Conclusions:**

Nutrition impact symptoms are the core symptoms for home‐hospice care cancer patients with a survival period of 1 year or below. Moreover, hospice cancer patients with short survival expectancies have greater inter‐symptom impact.

## 1 Introduction

China currently has the highest number of new cancer cases and deaths in the world [1]. Despite advances in cancer diagnosis and treatment, the prognosis of patients with advanced cancer remains challenging. Palliative care has become increasingly evident [2]. However, palliative‐care patients with advanced cancer frequently develop multiple symptoms [3, 4], with reports of some patients having more than five concurrent symptoms [5, 6]. Symptom clusters consisting of two or more interrelated and co‐occurring symptoms may or may not have a common cause [7]. Overlooking the complex relationships between coexisting symptoms can lead to their misinterpretation and mismanagement, thus affecting patient outcomes [8]. To enhance symptom management of cancer patients, a growing number of researchers have shifted their focus from examining single symptoms to investigating the intricacies and interplay among symptom clusters.

Previous studies have commonly used principal component analysis, hierarchical cluster analysis, and exploratory factor analysis to explore symptom clusters [9, 10, 11]. However, these studies only provide a broad view of the co‐occurrence of symptoms without explaining how they interact [12]. Hence, network analysis (NA) has recently been introduced to research on the management of cancer symptoms to describe and visualize the interactions between various symptoms and symptom clusters [13]. It can be used to conceptualize symptoms as interactions in the complex network of symptoms and explore changes in symptom clusters by comparing specific parameters of symptoms. Moreover, NA can quantify the importance of symptoms in the network using symptom mechanical indicators like centrality indicators [14, 15]. Thus, this approach helps identify core symptoms that activate other symptoms in the network and core symptom clusters [14].

Symptom clusters of cancer patients are specific to the patients' treatment stage [16, 17]. Studies on symptom networks in cancer patients mainly focus on patients receiving aggressive treatments [18, 19, 20]. However, symptom networks in patients with advanced cancer receiving hospice care have not yet been examined in detail. Since hospice patients no longer receive active medical treatment, it is not appropriate to directly apply the results of core symptom clusters from treatment‐stage patients to guide symptom management in the hospice care setting, particularly for those receiving care at home. The lack of clarity on which symptoms are the core symptoms for patients with advanced cancer in hospice care impacts symptom management. Therefore, this study sought to identify the core symptom clusters of hospice‐care cancer patients with different survival expectancies and compare the distinct features of their symptom networks.

## 2 Methods

### 2.1 Study Design and Setting

We conducted secondary data analysis using patients' records from the Fujian Provincial Hospital Hospice Center in Fuzhou, China. The center provides home‐based hospice care for patients with advanced cancer. Patients' records were obtained from January 2001 to December 2020 and included full assessments of patients during their first home visit and all subsequent follow‐ups until the termination of service or patient death.

### 2.2 Participants

Patients were included in this study according to the following criteria: (1) advanced cancer diagnosis and (2) < 12 months of survival expectancy assessed by clinical professionals. A total of 6946 patients were included, of which 6391 had < 6 months and 555 had 6–12 months of survival expectancy.

## 3 Measures

### 3.1 Sociodemographic and Clinical Characteristics

Patients' sociodemographic and clinical characteristics were recorded by hospice nurses during the first home visits and subsequent follow‐ups. The sociodemographic characteristics included sex, age, education level, presence of a spouse, number of children, source of income, monthly income, and awareness of the disease (full understanding, partial understanding, or complete ignorance). Additionally, the disease‐related clinical characteristics included cancer diagnosis, metastasis, previous cancer treatment, and previous analgesic treatment (no analgesic, bad, average, or satisfied). Survival expectancy was calculated as the number of days from the patient's first admission to death.

### 3.2 Self‐Reported Symptoms

We obtained patients' self‐reported symptoms recorded by hospice nurses to assess the symptom clusters. Before admission to the hospice service, a nurse visited each patient at home and assessed their symptoms according to a 40‐symptom list.

### 3.3 Data Analysis

All statistical analyses were conducted using R 4.3.1. All missing data were input using the MissForest algorithm [21]. Frequencies, percentages, means, and SDs were used to describe the sociodemographic informations, clinical characteristics, and symptom occurrence.

### 3.4 Symptom Selection

Symptoms with occurrence rates below 1% or above 99% were excluded before the symptom network construction to ensure variable consistency and mitigate minor variations. Random survival forest models were used to calculate the importance of each symptom related to survival expectancy, and 36 symptoms were selected for network analysis [22].

### 3.5 Network Estimations

Networks were constructed using the R packages “qgraph” and “IsingFit,” which combine L1‐regularized logistic regression with model selection. Based on the extended Bayesian information criterion, these packages are appropriate for our binary data [23]. We applied eLasso with AND‐rule and *γ* = 0.25 to obtain a sparse and, therefore, interpretable network [24]. In symptom networks, each node represented one symptom and edges indicated the magnitude of the association (the logistic regression coefficient) between two nodes. Undirected weighted association networks were constructed using the Fruchterman–Reingold algorithm and Spring layout [25]. To calculate the density of different networks, we used ∑*s* to indicate the strength of the symptom network interconnection, which is the absolute value of all Spearman coefficients between two nodes and can be recognized as a crucial indicator [26, 27].

### 3.6 Centrality Analysis and Community Detection

Three node centrality indices (strength, betweenness, and closeness) were calculated to evaluate the importance of each symptom within the network [28]. The symptom with the highest centrality index was taken as the core symptom in the network, and the symptom cluster where the core symptom was located was recognized as the core symptom cluster [29]. The Walktrap algorithm was used to identify symptom clusters, which were named according to previous studies [30]. All centrality indices were standardized (reported by *r*‐values between 0 and 1) to ensure that nodes were comparable.

### 3.7 Network Accuracy and Stability

The accuracy of estimated networks was evaluated in two ways. The accuracy of the edge weights was determined by calculating 95% confidence intervals (CI) and observing the differences in strength between the original and the generated dataset [31]. The stability of centrality indices was determined by the correlation stability coefficient (CS coefficient). CS coefficients above 0.25 indicate moderate stability, while those above 0.5 demonstrate high stability [28]. Bootstrapped difference tests were also performed to measure the difference in centrality indices and edge weights between nodes. Significance levels were reported at *p* < 0.05.

### 3.8 Network Comparison Test

To detect between‐group network differences, network comparison tests were conducted using the package “Network Comparison Test,” which is based on the estimation of several invariance measures, including the network structure, global strength, and edge strength [32]. However, network comparison tests are less accurate if there are different sample sizes between two subgroups [33]. To ensure the subgroup sample sizes were equal, a sample was randomly selected from the larger (0–6 month) group to match the size of the smaller (6–12 month) group. The results of the network comparison test were presented as averaged invariance statistics and *p* values.

### 3.9 Ethics Approval and Consent to Participate

This study was based on a secondary analysis of case records in a hospice center of Fujian Provincial Hospital. We obtained study approval from the Ethics Committee of Fujian Medical University (Authorization no. 2022–91), which decided that written informed consent from the participants was unnecessary and waived the need for informed consent because of the retrospective nature of the study. All methods were carried out in accordance with relevant guidelines and regulations.

## 4 Results

### 4.1 Characteristics of Participants

Of the 6946 patients, 40.74% were men and 59.26% were women. The mean age of patients was 62.20 ± 12.86 years, and 58.36% were older than 60 years. Details regarding participant characteristics are shown in Table S1.

### 4.2 Symptom Occurrence

Table 1 Illustrates the occurrence of symptoms in patients. Among the symptoms occurring in over 50% of cases, weight loss (95.02%) was the most common, followed by loss of appetite (62.04%), insomnia (59.17%), and malnutrition (55.60%). The incidence of weight loss, insomnia, edema, excessive sweating, loss of appetite, hiccups, reflux/belching, swallowing obstruction, nausea, vomiting, constipation, abdominal distension, stomachache, coughing up sputum, oliguria, unsteady gait, impaired mobility, cachexia, and ascites was significantly different among patients with a survival expectancy of < 6 months and those with a survival expectancy of > 6 months (*p* < 0.05).

**TABLE 1:** Symptom occurrence (*n*, %).

Symptom	Code	Total (*n* = 6946)	Survival expectancy		*p*
			< 6 months (*n* = 6391)	6–12 months (*n* = 555)	
Weight loss	S1	6600 (95.02)	6096 (95.38)	504 (90.81)	< 0.001
Insomnia	S2	4110 (59.17)	3804 (59.52)	306 (55.14)	0.049
Edema	S3	1843 (26.53)	1778 (27.82)	65 (11.71)	< 0.001
Excessive sweating	S4	1039 (14.96)	976 (15.27)	63 (11.35)	0.015
Loss of appetite	S5	4309 (62.04)	4007 (62.7)	302 (54.41)	< 0.001
Hiccups	S6	396 (5.70)	380 (5.95)	16 (2.88)	0.004
Reflux/belching	S7	509 (7.33)	486 (7.60)	23 (4.14)	0.004
Swallowing obstruction	S8	787 (11.33)	760 (11.89)	27 (4.86)	< 0.001
Nausea	S9	2298 (33.08)	2180 (34.11)	118 (21.26)	< 0.001
Vomiting	S10	2200 (31.67)	2070 (32.39)	130 (23.42)	< 0.001
Constipation	S11	3431 (49.4)	3205 (50.15)	226 (40.72)	< 0.001
Abdominal distension	S12	2213 (31.86)	2110 (33.02)	103 (18.56)	< 0.001
Stomachache	S13	3416 (49.18)	3220 (50.38)	196 (35.32)	< 0.001
Gastrointestinal bleeding	S14	224 (3.22)	213 (3.33)	11 (1.98)	0.109
Cough	S15	2091 (30.1)	1941 (30.37)	150 (27.03)	0.110
Coughing up sputum	S16	1435 (20.66)	1355 (21.2)	80 (14.41)	< 0.001
Gasping	S17	704 (10.14)	657 (10.28)	47 (8.47)	0.199
Chest pain	S18	1610 (23.18)	1477 (23.11)	133 (23.96)	0.686
Hemoptysis	S19	309 (4.45)	288 (4.51)	21 (3.78)	0.494
Chest tightness	S20	1028 (14.80)	955 (14.94)	73 (13.15)	0.282
Palpitation	S21	257 (3.70)	241 (3.77)	16 (2.88)	0.344
Orthopnea	S22	87 (1.25)	79 (1.24)	8 (1.44)	0.827
Dysuria	S23	236 (3.40)	214 (3.35)	22 (3.96)	0.519
Urinary incontinence	S24	160 (2.30)	151 (2.36)	9 (1.62)	0.333
Oliguria	S25	301 (4.33)	292 (4.57)	9 (1.62)	0.002
Arthralgia	S26	889 (12.8)	808 (12.64)	81 (14.59)	0.21
Dizziness	S27	1443 (20.77)	1343 (21.01)	100 (18.02)	0.106
Headache	S28	1022 (14.71)	952 (14.9)	70 (12.61)	0.163
Unsteady gait	S29	2127 (30.62)	1979 (30.97)	148 (26.67)	0.039
Hearing loss	S30	487 (7.01)	437 (6.84)	50 (9.01)	0.067
Vision loss	S31	267 (3.84)	246 (3.85)	21 (3.78)	1
Impaired mobility	S32	434 (6.25)	423 (6.62)	11 (1.98)	< 0.001
Malnutrition	S33	3862 (55.60)	3561 (55.72)	301 (54.23)	0.528
Cachexia	S34	676 (9.73)	653 (10.22)	23 (4.14)	< 0.001
Pleural effusion	S35	351 (5.05)	325 (5.09)	26 (4.68)	0.755
Ascites	S36	633 (9.11)	620 (9.70)	13 (2.34)	< 0.001

### 4.3 Network Estimation

**FIGURE 1:**
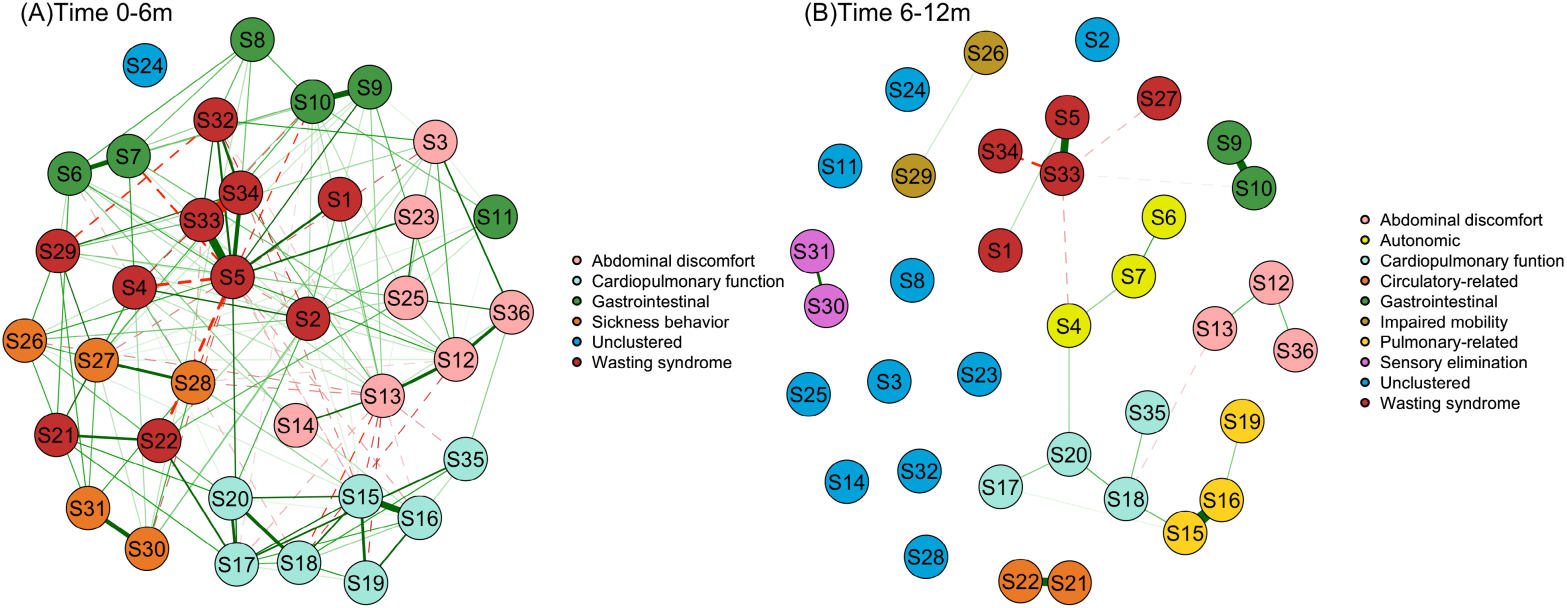
Estimated symptom networks and symptom clusters. Solid lines indicate positive relationships, dashed lines indicate negative relationships, and thick lines indicate strong connections between nodes. Node colors represent communities detected in each network.

The network structures of symptoms for patients with different survival expectancies are shown in Figure 1. The network density of symptoms for patients with a survival expectancy of < 6 months was 91.99. The top pair with the strongest positive connections was identified as malnutrition loss of appetite. Conversely, a substantial negative connection was observed between malnutrition and cachexia. The network density for patients with survival expectancies of 6–12 months was 28.39. Coughing was most positively associated with phlegm, followed by loss of appetite–malnutrition and nausea–vomiting. Malnutrition had the strongest negative associations with hyperhidrosis, dizziness, and cachexia (Table S2).

### 4.4 Centrality Indices

**FIGURE 2:**
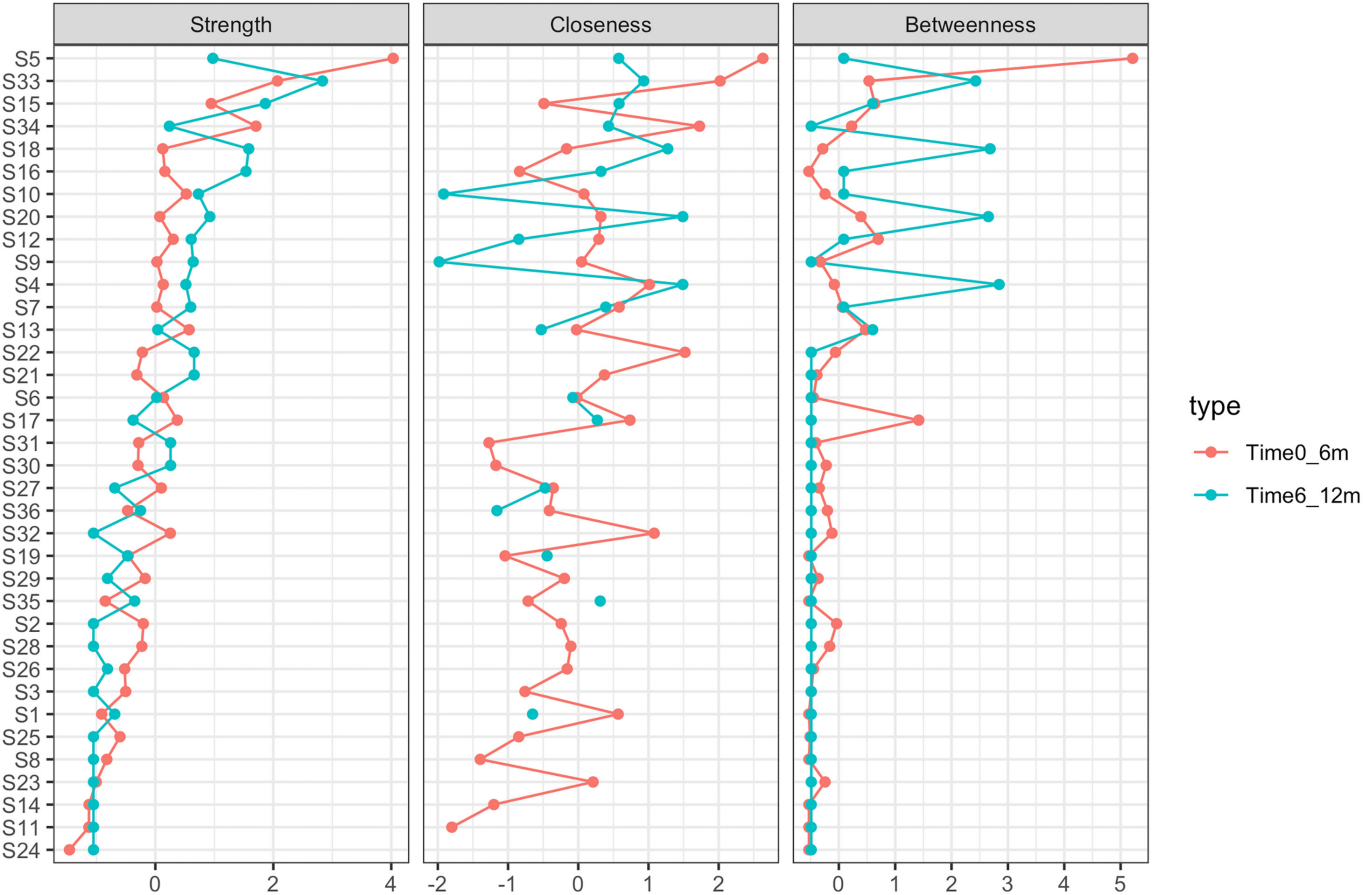
Comparison of network centrality indices. Centrality indices (strength, closeness, and betweenness) are shown for all symptoms in the networks (*z*‐scores).

The standardized estimates of the centrality indices (i.e., strength, closeness, and betweenness) for the symptom networks of different survival expectancies are presented in Figure 2. Specifically, in the 0‐ to 6‐month network, loss of appetite had the highest strength centrality (rs = 4.03, rb = 5.21, rc = 2.63). In the 6‐ to 12‐month network, the symptom with the highest strength centrality score was malnutrition (rs = 2.83, rb = 2.43, rc = 0.93). Details of other centrality indices are shown in Table S3.

### 4.5 Community Detection

**FIGURE 3:**
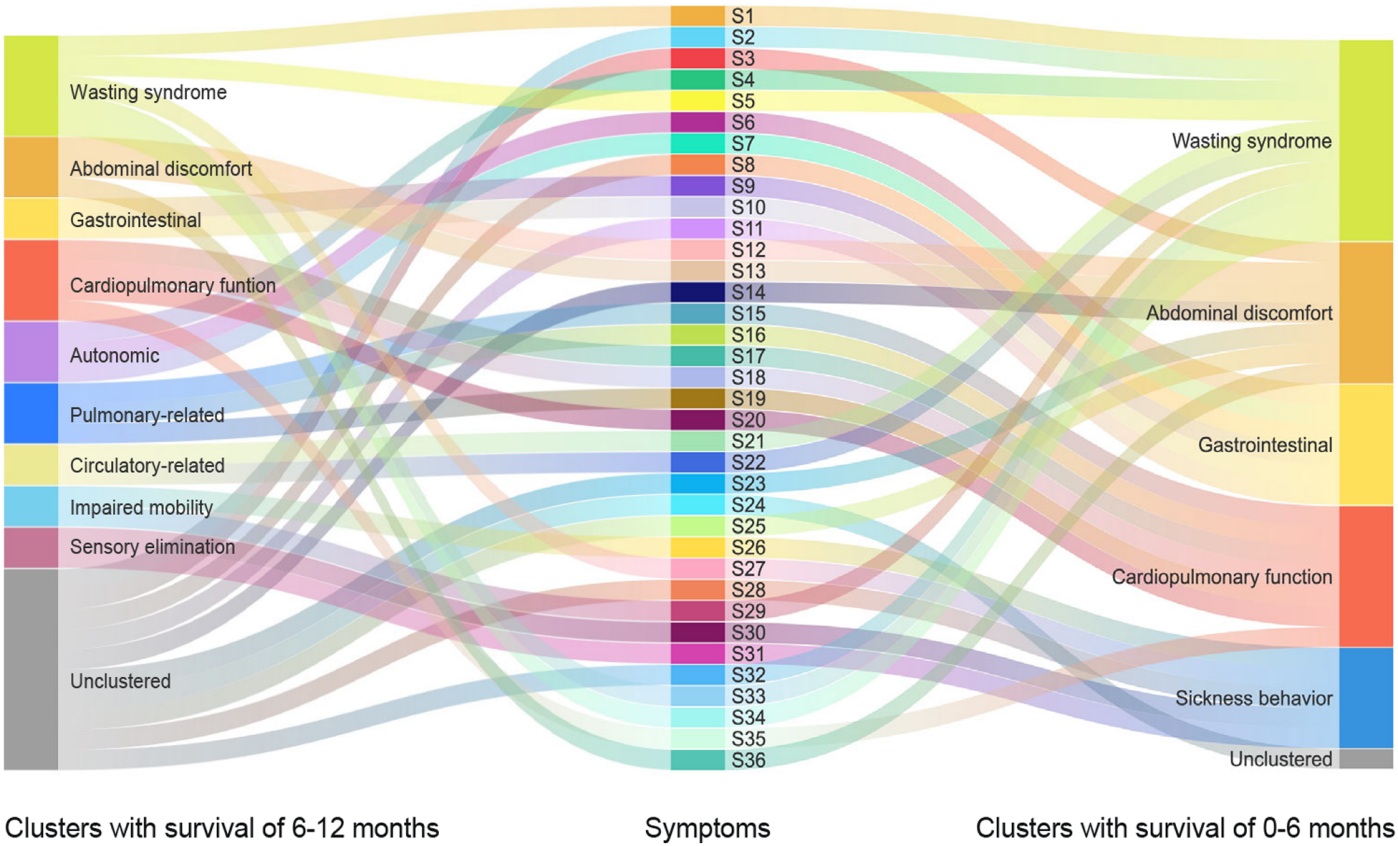
Trajectories of change in symptom clusters.

Figure 1 Illustrates the symptom clusters for patients with different survival expectancies. Five symptom clusters were identified in the symptom network for patients with < 6 months of survival expectancy. Nine symptom clusters were identified in the symptom network for patients who survived for 6–12 months. The composition of symptoms within the different symptom clusters varied across the networks. Details regarding the variations are shown in Table S4 and Figure 3.

### 4.6 Network Accuracy and Stability

The bootstrapped 95% CI of edge weights is shown in Figure S1. The bootstrap CI for both groups indicated that these two networks were accurate. Additionally, the stability of strength centrality was higher than “closeness” and “betweenness.” The CS coefficient was 0.75 in the network of patients with survival expectancies of < 6 months and 0.28 in patients with survival expectancies of 6–12 months (Figure 2).

### 4.7 Network Comparison Test

We compared the structure of symptom networks, centrality indices, and network densities among patients with different survival expectancies. While no significant difference was observed in the network structure between the two groups (*p* = 0.471), the global strength invariance test revealed a significant difference (*p* = 0.024). By comparing the network centrality indices between the two groups, we identified the differences in symptom centrality over survival expectancy. Notably, loss of appetite exhibited increased prominence in terms of the strength, closeness, and betweenness centralities (Figure 2).

## 5 Discussion

In a large sample of heterogeneous hospice patients with advanced cancer, we used NA to visualize the core symptom clusters and the structure of symptom network in patients living with expected survival expectancies of 6–12 months and < 6 months. We also assessed the difference of core symptoms and symptom clusters with different life expectancy. In this study, we found that people living with < 6 months of survival expectancy have a higher density of symptom networks than those with 6–12 months of survival expectancy. Notably, the nutrition impact symptoms (NIS) were the most notable symptom clusters in both groups.

In patients with survival expectancies of 6–12 months and < 6 months, loss of appetite and malnutrition were the core symptoms. Interestingly, these two symptoms were strongly connected and were categorized into the NIS through community detection. This is consistent with the findings conducted by Papachristou et al. [18]. Because of the high catabolic and low anabolic state due to tumor progression and anti‐tumor therapy, the centrality of NIS in the symptom network is inevitable [34, 35]. However, differences in core symptoms in patients with different survival expectancies were identified. We compared the centrality levels of nodes in patients with different survival stages and found that some symptoms changed with the decline of survival expectancy, reflecting the dynamic and complex nature of the symptom experience in patients with advanced cancer. Notably, the importance of decreased appetite increased the closer patients were to death. Appetite disorders are highly prevalent in people living with advanced cancer and significantly impact patients' nutritional status as cancer progresses [36, 37]. Loss of appetite is not only a critical factor in the development of malignant fluid but also its primary manifestation [38]. Moreover, loss of appetite does not occur independently but is often reported in conjunction with other symptoms such as pain or fatigue, and it significantly impacts the quality of life (QOL) of patients [39].

Using community detection, we explored the number and composition of symptom clusters in patients with advanced cancer receiving home hospice care. We identified nine symptom clusters in the symptom networks of patients with 6–12 months of survival expectancy and five symptom clusters in patients with < 6 months of survival expectancy. Compared to patients with 6–12 months of survival expectancy, certain symptom clusters in patients with < 6 months of survival expectancy tended to contain more symptoms. This finding indicating a more focused and pronounced symptom experience in terminally ill patients compared to patients with longer survival expectancy.

Through the definition of core symptom cluster, the core symptom cluster in both symptom networks was identified as the “wasting syndrome cluster,” consisting of loss of appetite, malnutrition, weight loss, cachexia, and weakness. Simão et al. identified three symptom clusters in patients with advanced cancer, among which a distinct NIS symptom cluster was presented [40]. Jimenez et al. found that two of the four symptom clusters in patients with advanced cancer were NIS [41]. In another exploration of symptom clusters for patients with cancer, Xiao et al. found that the NIS was the most severe of the four identified [9]. Importantly, the NIS is the most common and severe cluster reported in people living with advanced cancer [42, 43]. Hence, the results of the present study align with these studies.

In our study, the wasting syndrome cluster in patients with < 6 months of survival expectancy tended to contain more weakness‐manifested symptoms than those with 6–12 months of survival expectancy. This finding suggests that the negative effects of nutritional status become more pronounced as the patient nears the end of their life [44]. The wasting syndrome cluster represents a common and severe condition in patients with advanced cancer in the later stages of life. Similarly, more than 50% of patients with advanced cancer are diagnosed with cancer anorexia–cachexia syndrome, which is considered a complex multifactorial syndrome characterized by loss of appetite, involuntary weight loss, and loss of skeletal muscle that can directly lead to poor prognosis and low QOL [45, 46, 47].

Network density and global strength were examined to reflect the overall symptom burden and prognosis of the cancer patients from a symptom perspective. Comparing a series of mechanical indicators showed that the density and global strength of the network were higher in patients with < 6 months of survival expectancy than in those with survival expectancies of 6–12 months. This finding aligns with results reported by Zhu et al. [25], who reported a significantly higher network density in patients with < 5 years of survival expectancy compared to those with ≥ 5 years. High network density and global strength reflected high levels of symptom distress, disturbance, and complexity, which may be detrimental to QOL and survival in such patients [13, 48]. Our results indicated strong connections between symptoms and symptom clusters in patients with shorter survival expectancies. Therefore, changes in one symptom may induce severe changes in the connected symptoms in the overall network. In other words, the symptom network may exhibit higher entropy in people with shorter survival expectancy, indicating the whole symptom network is more unstable and vulnerable, with more chaotic connections between symptoms [49, 50].

Given the high occurrence and impact of NIS in advanced cancer patients, these types of symptoms should be considered important components of routine advanced cancer management. Because they reduce patient survival expectancies, the distress caused cannot be ignored—especially for terminal cancer patients who need detailed symptom assessments and interventions [51]. It is imperative that we address these core NIS through a comprehensive approach that integrates palliative, supportive, and nutritional care. By providing evidence‐based, non‐aggressive interventions to manage these core NIS and offering timely nutritional support, healthcare teams can alleviate distressing symptoms and potentially prevent or delay the progression of cachexia [52]. This aligns with the primary focus of hospice care on symptom control, without causing additional distress to the patient [53]. Such an integrated approach may be a key strategy for improving overall symptom burden, QOL, and even life expectancy in hospice patients with advanced cancer.

### 5.1 Strength and Limitations

To our knowledge, this is the first study using NA to explore symptom networks for Chinese patients with advanced cancer receiving home‐based hospice care that employs a large sample size to offer sufficient statistical validity to provide evidence of core and complex symptom clusters. Our study offers fresh insights into clinical practice regarding core symptoms in hospice patients with advanced cancer and promotes our understanding of the structural relationships between co‐morbidities and symptom clusters in these patients.

Several limitations must be noted. First, the design of this study and the method of sample selection limits the inference of causality among symptoms. Future research should use longitudinal data to validate these dynamic networks and investigate possible changes in core symptoms over longer life expectancies in patients with advanced cancer. Second, this study only included home‐based hospice patients with advanced cancer from a single center, which is not fully representative of patients with advanced cancer who are of different ethnicities from different regions. This limitation may have introduced selection bias and affected the generalizability of the findings. It is necessary to conduct multicenter studies to validate the reliability and generalizability of the results. Third, we only used prevalence data of patients' self‐reported symptoms and did not include other symptom‐experience data, such as levels of symptom severity and distress. This may have introduced information and subjectivity bias and affected the generalizability of the findings. Multidimensional symptom‐experience data and comparisons of symptom clusters with objective indicators are needed to better understand how they might vary across symptom dimensions.

## 6 Conclusion

Hospice cancer patients with shorter survival expectancies have a greater inter‐symptom impact and burden. These NIS were identified as the core symptom cluster; therefore, loss of appetite should be a core management target for intervention. This study emphasizes the need to address multiple symptom burdens in cancer patients through comprehensive symptom assessment and management, particularly in NIS management strategies.

## Author Contributions

**Yitao Wei:** data curation (equal), writing – original draft (equal). **Wan Cheng:** data curation (equal), writing – original draft (equal). **Yuanfeng Lu:** data curation (equal), writing – original draft (equal). **Zheng Zhu:** methodology (equal), writing – review and editing (equal). **Guiru Xu:** software (equal). **Hong Wu:** supervision (equal). **Shaowei Lin:** software (equal). **Huimin Xiao:** conceptualization (equal), project administration (equal), writing – review and editing (equal).

## Conflicts of Interest

The authors declare no conflicts of interest.

## Supporting information


AppendixS1.

## Data Availability

The sharing of data is restricted by existing agreements, thus preventing the dataset from being made publicly available. Access to the data may be granted to individuals who meet pre‐determined criteria for confidential access. The statistical analysis code is available upon request from the corresponding author.
