# Supplementary material for: Features and Differences in Core Symptom Clusters in Home‐Based Hospice Patients With Advanced Cancer: A Network Analysis
Source: Cancer Med. 2024 Nov 4;13(21):e70370. doi: 10.1002/cam4.70370 (PMC11532887; doi:10.1002/cam4.70370)
Supplement: Supplementary file 1 — AppendixS1. [file CAM4-13-e70370-s001.docx]

Supplementary materials

Table S1 Participant Characteristics (n, %)

| Characteristic | Total  (n = 6946) | Survival expectancy | | P |
| --- | --- | --- | --- | --- |
|  |  | <6 months  (n = 6391) | 6–12 months  (n = 555) |  |
| Sex | | | | <0.001 |
| Male | 2830 (40.74) | 2555 (39.98) | 275 (49.55) |  |
| Female | 4116 (59.26) | 3836 (60.02) | 280 (50.45) |  |
| Age (years) | | | | 0.933 |
| <60 | 2873 (41.36) | 2642 (41.34) | 231 (41.62) |  |
| ≥60 | 4073 (58.64) | 3749 (58.66) | 324 (58.38) |  |
| Spouse | | | | 0.376 |
| Married | 4203 (60.51) | 3865 (60.48) | 338 (60.90) |  |
| Unmarried | 110 (1.58) | 98 (1.53) | 12 (2.16) |  |
| Divorced | 135 (1.94) | 129 (2.02) | 6 (1.08) |  |
| Widowed | 688 (9.90) | 638 (9.98) | 50 (9.01) |  |
| NA | 1810 (26.06) | 1661 (25.99) | 149 (26.85) |  |
| Place of residence | | | | 0.025 |
| Rural | 3676 (52.92) | 3408 (53.32) | 268 (48.29) |  |
| Urban | 3270 (47.08) | 2983 (46.68) | 287 (51.71) |  |
| Education level | | | | 0.168 |
| No formal education | 1078 (15.52) | 975 (15.26) | 103 (18.56) |  |
| Primary school | 3490 (50.24) | 3229 (50.52) | 261 (47.03) |  |
| Junior high school | 1567 (22.56) | 1444 (22.59) | 123 (22.16) |  |
| Senior high school or above | 811 (11.68) | 743 (11.63) | 68 (12.25) |  |
| Tumor type | | | | <0.001 |
| Lung cancer | 1542 (22.2) | 1395 (21.83) | 147 (26.49) |  |
| Gastric cancer | 1486 (21.39) | 1408 (22.03) | 78 (14.05) |  |
| Liver cancer | 1002 (14.43) | 938 (14.68) | 64 (11.53) |  |
| Laryngeal carcinoma | 750 (10.8) | 670 (10.48) | 80 (14.41) |  |
| Breast cancer | 295 (4.25) | 262 (4.10) | 33 (5.95) |  |
| Esophageal carcinoma | 260 (3.74) | 245 (3.83) | 15 (2.70) |  |
| Pancreatic cancer | 254 (3.66) | 239 (3.74) | 15 (2.70) |  |
| Nasopharyngeal cancer | 230 (3.31) | 212 (3.32) | 18 (3.24) |  |
| Cervical cancer | 149 (2.15) | 133 (2.08) | 16 (2.88) |  |
| Other | 978 (14.08) | 889 (13.91) | 89 (16.04) |  |
| Metastasis |  |  |  | 0.058 |
| Yes | 6147 (88.5) | 5670 (88.72) | 477 (85.95) |  |
| No | 799 (11.5) | 721 (11.28) | 78 (14.05) |  |
| Medical insurance |  |  |  | 0.814 |
| Yes | 6665 (95.95) | 6134 (95.98) | 531 (95.68) |  |
| No | 281 (4.05) | 257 (4.02) | 24 (4.32) |  |
| Previous surgery |  |  |  | 0.053 |
| Yes | 2534 (36.48) | 2310 (36.14) | 224 (40.36) |  |
| No | 4412 (63.52) | 4081 (63.86) | 331 (59.64) |  |
| Previous chemotherapy (Yes/No) |  |  |  | 0.030 |
| Yes | 2882 (41.49) | 2627 (41.10) | 255 (45.95) |  |
| No | 4064 (58.51) | 3764 (58.90) | 300 (54.05) |  |
| Previous radiotherapy (Yes/No) |  |  |  | <0.001 |
| Yes | 1116 (16.07) | 999 (15.63) | 117 (21.08) |  |
| No | 5830 (83.93) | 5392 (84.37) | 438 (78.92) |  |
| Diabetic comorbidity (Yes/No) |  |  |  | 0.270 |
| Yes | 548 (7.89) | 497 (7.78) | 51 (9.19) |  |
| No | 6398 (92.11) | 5894 (92.22) | 504 (90.81) |  |
| Hypertension comorbidity (Yes/No) |  |  |  | 0.596 |
| Yes | 1238 (17.82) | 1134 (17.74) | 104 (18.74) |  |
| No | 5708 (82.18) | 5257 (82.26) | 451 (81.26) |  |
| Previous tobacco and alcohol intake |  |  |  | 0.101 |
| Yes | 1459 (21.00) | 1358 (21.25) | 101 (18.20) |  |
| No | 5487 (79.00) | 5033 (78.75) | 454 (81.80) |  |

|  | S1 | S2 | S3 | S4 | S5 | S6 | S7 | S8 | S9 | S10 | S11 | S12 | S13 | S14 | S15 | S16 | S17 | S18 | S19 | S20 | S21 | S22 | S23 | S24 | S25 | S26 | S27 | S28 | S29 | S30 | S31 | S32 | S33 | S34 | S35 | S36 |
| --- | --- | --- | --- | --- | --- | --- | --- | --- | --- | --- | --- | --- | --- | --- | --- | --- | --- | --- | --- | --- | --- | --- | --- | --- | --- | --- | --- | --- | --- | --- | --- | --- | --- | --- | --- | --- |
| S1 | 0.00 | 0.40 | 0.00 | 0.00 | 1.16 | 0.00 | 0.00 | 0.00 | 0.00 | 0.00 | 0.00 | 0.00 | 0.36 | 0.00 | 0.00 | 0.00 | 0.00 | 0.00 | 0.00 | 0.00 | 0.00 | 0.00 | 0.00 | 0.00 | 0.00 | 0.00 | 0.00 | 0.00 | 0.00 | 0.00 | 0.00 | 0.00 | 0.00 | 0.00 | 0.00 | 0.00 |
| S2 | 0.40 | 0.00 | 0.00 | 0.71 | -0.28 | 0.14 | 0.00 | 0.00 | 0.13 | 0.18 | 0.49 | 0.00 | 0.00 | 0.00 | 0.08 | 0.00 | 0.00 | 0.00 | 0.00 | 0.46 | 0.00 | 0.00 | 0.00 | 0.00 | 0.22 | 0.34 | 0.07 | 0.00 | 0.06 | 0.32 | 0.00 | -0.50 | -0.02 | 0.00 | 0.00 | 0.00 |
| S3 | 0.00 | 0.00 | 0.00 | 0.00 | -0.39 | 0.00 | 0.00 | 0.00 | 0.06 | 0.00 | 0.07 | 0.48 | 0.04 | 0.00 | 0.00 | 0.00 | 0.00 | 0.00 | 0.00 | 0.00 | 0.00 | 0.22 | 0.00 | 0.00 | 0.26 | 0.00 | 0.00 | 0.00 | 0.23 | 0.19 | 0.00 | 0.55 | 0.00 | 0.00 | 0.00 | 0.84 |
| S4 | 0.00 | 0.71 | 0.00 | 0.00 | -1.41 | 0.00 | 0.56 | 0.00 | 0.00 | 0.25 | 0.00 | 0.00 | 0.16 | 0.00 | 0.00 | 0.18 | 0.00 | 0.00 | 0.00 | 0.06 | 0.52 | 0.00 | 0.00 | 0.00 | 0.00 | 0.14 | 0.48 | 0.38 | 0.00 | 0.22 | 0.00 | 0.00 | -0.51 | 0.00 | 0.00 | 0.00 |
| S5 | 1.16 | -0.28 | -0.39 | -1.41 | 0.00 | 0.48 | -1.07 | 0.00 | 0.66 | -0.67 | 0.00 | 0.55 | 0.00 | 0.00 | -0.21 | -0.22 | 0.69 | 0.00 | 0.00 | 0.00 | 0.00 | -1.44 | 0.92 | 0.00 | 0.00 | 0.52 | -0.30 | -0.58 | 0.11 | -0.36 | 0.00 | 1.11 | 4.09 | 2.04 | 0.00 | 0.00 |
| S6 | 0.00 | 0.14 | 0.00 | 0.00 | 0.48 | 0.00 | 2.24 | 0.48 | 0.23 | 0.29 | 0.00 | 0.14 | 0.17 | 0.32 | 0.00 | 0.00 | 0.00 | -0.16 | 0.00 | 0.00 | 0.50 | 0.00 | 0.00 | 0.00 | 0.00 | 0.00 | 0.00 | 0.00 | 0.18 | 0.00 | 0.00 | 0.27 | 0.00 | 0.00 | 0.00 | 0.00 |
| S7 | 0.00 | 0.00 | 0.00 | 0.56 | -1.07 | 2.24 | 0.00 | 0.37 | 0.10 | 0.36 | 0.00 | 0.37 | 0.13 | 0.00 | 0.00 | 0.00 | 0.00 | 0.00 | 0.00 | 0.00 | 0.00 | 0.00 | 0.00 | 0.00 | 0.00 | 0.00 | 0.00 | 0.00 | 0.00 | 0.00 | 0.00 | 0.00 | 0.00 | 0.00 | 0.00 | 0.00 |
| S8 | 0.00 | 0.00 | 0.00 | 0.00 | 0.00 | 0.48 | 0.37 | 0.00 | 0.00 | 0.44 | 0.00 | 0.00 | 0.00 | 0.00 | 0.00 | 0.00 | 0.00 | 0.00 | 0.00 | 0.00 | 0.00 | 0.00 | 0.00 | 0.00 | 0.00 | 0.00 | 0.00 | 0.00 | 0.00 | 0.17 | 0.00 | 0.40 | 0.00 | 0.36 | 0.00 | 0.00 |
| S9 | 0.00 | 0.13 | 0.06 | 0.00 | 0.66 | 0.23 | 0.10 | 0.00 | 0.00 | 3.05 | 0.00 | 0.44 | 0.26 | 0.00 | 0.00 | 0.00 | 0.00 | 0.00 | 0.00 | 0.00 | 0.00 | 0.00 | 0.00 | 0.00 | 0.00 | 0.00 | 0.12 | 0.00 | 0.00 | 0.00 | 0.00 | 0.00 | 0.00 | 0.16 | 0.00 | 0.00 |
| S10 | 0.00 | 0.18 | 0.00 | 0.25 | -0.67 | 0.29 | 0.36 | 0.44 | 3.05 | 0.00 | 0.35 | 0.00 | 0.21 | 0.00 | 0.07 | 0.00 | -0.09 | 0.00 | 0.00 | 0.00 | 0.00 | 0.00 | 0.00 | 0.00 | 0.17 | 0.00 | 0.46 | 0.00 | 0.00 | 0.00 | 0.00 | 0.00 | -0.36 | 0.00 | 0.00 | 0.00 |
| S11 | 0.00 | 0.49 | 0.07 | 0.00 | 0.00 | 0.00 | 0.00 | 0.00 | 0.00 | 0.35 | 0.00 | 0.03 | 0.00 | 0.00 | 0.00 | 0.00 | 0.00 | 0.00 | 0.00 | 0.00 | 0.00 | 0.00 | 0.00 | 0.00 | 0.13 | 0.00 | 0.09 | 0.00 | 0.00 | 0.00 | 0.00 | 0.00 | 0.00 | 0.00 | 0.00 | 0.00 |
| S12 | 0.00 | 0.00 | 0.48 | 0.00 | 0.55 | 0.14 | 0.37 | 0.00 | 0.44 | 0.00 | 0.03 | 0.00 | 1.59 | 0.00 | 0.00 | 0.00 | 0.00 | -0.44 | 0.00 | 0.00 | 0.00 | 0.00 | 0.00 | 0.00 | 0.20 | 0.00 | 0.09 | -0.11 | 0.15 | 0.00 | 0.00 | 0.00 | 0.00 | 0.00 | 0.00 | 1.59 |
| S13 | 0.36 | 0.00 | 0.04 | 0.16 | 0.00 | 0.17 | 0.13 | 0.00 | 0.26 | 0.21 | 0.00 | 1.59 | 0.00 | 0.85 | -0.43 | -0.12 | -0.19 | -0.62 | -0.50 | 0.00 | 0.00 | 0.00 | 0.00 | 0.00 | 0.00 | -0.28 | 0.00 | -0.28 | 0.00 | 0.00 | 0.00 | -0.13 | 0.00 | 0.41 | -0.22 | 0.16 |
| S14 | 0.00 | 0.00 | 0.00 | 0.00 | 0.00 | 0.32 | 0.00 | 0.00 | 0.00 | 0.00 | 0.00 | 0.00 | 0.85 | 0.00 | 0.00 | 0.00 | 0.00 | 0.00 | 0.00 | 0.00 | 0.00 | 0.00 | 0.00 | 0.00 | 0.00 | 0.00 | 0.00 | 0.00 | 0.00 | 0.00 | 0.00 | 0.00 | 0.00 | 0.00 | 0.00 | 0.00 |
| S15 | 0.00 | 0.08 | 0.00 | 0.00 | -0.21 | 0.00 | 0.00 | 0.00 | 0.00 | 0.07 | 0.00 | 0.00 | -0.43 | 0.00 | 0.00 | 3.19 | 0.65 | 0.86 | 1.41 | 0.74 | 0.00 | 0.00 | 0.00 | 0.00 | 0.00 | 0.09 | 0.00 | 0.27 | 0.00 | 0.00 | 0.00 | -0.30 | -0.12 | 0.00 | 0.00 | 0.00 |
| S16 | 0.00 | 0.00 | 0.00 | 0.18 | -0.22 | 0.00 | 0.00 | 0.00 | 0.00 | 0.00 | 0.00 | 0.00 | -0.12 | 0.00 | 3.19 | 0.00 | 0.50 | 0.26 | 0.91 | 0.06 | 0.00 | 0.00 | 0.00 | 0.00 | 0.00 | 0.00 | 0.00 | 0.04 | 0.00 | 0.00 | 0.00 | 0.00 | 0.00 | 0.00 | 0.19 | 0.00 |
| S17 | 0.00 | 0.00 | 0.00 | 0.00 | 0.69 | 0.00 | 0.00 | 0.00 | 0.00 | -0.09 | 0.00 | 0.00 | -0.19 | 0.00 | 0.65 | 0.50 | 0.00 | 0.41 | 0.10 | 1.36 | 0.55 | 0.96 | 0.00 | 0.00 | 0.00 | 0.00 | 0.00 | 0.00 | 0.00 | 0.00 | 0.00 | 0.00 | 0.00 | 0.00 | 0.90 | 0.00 |
| S18 | 0.00 | 0.00 | 0.00 | 0.00 | 0.00 | -0.16 | 0.00 | 0.00 | 0.00 | 0.00 | 0.00 | -0.44 | -0.62 | 0.00 | 0.86 | 0.26 | 0.41 | 0.00 | 0.57 | 1.69 | 0.00 | 0.00 | 0.00 | 0.00 | 0.00 | 0.00 | 0.00 | 0.00 | 0.00 | 0.00 | 0.00 | 0.00 | 0.00 | 0.00 | 0.54 | 0.00 |
| S19 | 0.00 | 0.00 | 0.00 | 0.00 | 0.00 | 0.00 | 0.00 | 0.00 | 0.00 | 0.00 | 0.00 | 0.00 | -0.50 | 0.00 | 1.41 | 0.91 | 0.10 | 0.57 | 0.00 | 0.00 | 0.00 | 0.00 | 0.00 | 0.00 | 0.00 | 0.00 | 0.00 | 0.00 | 0.00 | 0.00 | 0.00 | 0.00 | 0.00 | 0.00 | 0.00 | 0.00 |
| S20 | 0.00 | 0.46 | 0.00 | 0.06 | 0.00 | 0.00 | 0.00 | 0.00 | 0.00 | 0.00 | 0.00 | 0.00 | 0.00 | 0.00 | 0.74 | 0.06 | 1.36 | 1.69 | 0.00 | 0.00 | 0.57 | 0.00 | 0.00 | 0.00 | 0.00 | 0.42 | 0.00 | 0.00 | 0.00 | 0.00 | 0.00 | 0.00 | 0.00 | 0.00 | 0.00 | 0.00 |
| S21 | 0.00 | 0.00 | 0.00 | 0.52 | 0.00 | 0.50 | 0.00 | 0.00 | 0.00 | 0.00 | 0.00 | 0.00 | 0.00 | 0.00 | 0.00 | 0.00 | 0.55 | 0.00 | 0.00 | 0.57 | 0.00 | 1.27 | 0.00 | 0.00 | 0.00 | 0.00 | 0.59 | 0.00 | 0.00 | 0.00 | 0.00 | 0.00 | 0.00 | 0.00 | 0.00 | 0.00 |
| S22 | 0.00 | 0.00 | 0.22 | 0.00 | -1.44 | 0.00 | 0.00 | 0.00 | 0.00 | 0.00 | 0.00 | 0.00 | 0.00 | 0.00 | 0.00 | 0.00 | 0.96 | 0.00 | 0.00 | 0.00 | 1.27 | 0.00 | 0.00 | 0.00 | 0.44 | 0.00 | 0.00 | 0.00 | 0.00 | 0.00 | 0.00 | 0.00 | 0.00 | 0.00 | 0.00 | 0.00 |
| S23 | 0.00 | 0.00 | 0.00 | 0.00 | 0.92 | 0.00 | 0.00 | 0.00 | 0.00 | 0.00 | 0.00 | 0.00 | 0.00 | 0.00 | 0.00 | 0.00 | 0.00 | 0.00 | 0.00 | 0.00 | 0.00 | 0.00 | 0.00 | 0.00 | 0.67 | 0.00 | 0.00 | 0.00 | 0.00 | 0.00 | 0.00 | 0.00 | 0.00 | 0.00 | 0.00 | 0.00 |
| S24 | 0.00 | 0.00 | 0.00 | 0.00 | 0.00 | 0.00 | 0.00 | 0.00 | 0.00 | 0.00 | 0.00 | 0.00 | 0.00 | 0.00 | 0.00 | 0.00 | 0.00 | 0.00 | 0.00 | 0.00 | 0.00 | 0.00 | 0.00 | 0.00 | 0.00 | 0.00 | 0.00 | 0.00 | 0.00 | 0.00 | 0.00 | 0.00 | 0.00 | 0.00 | 0.00 | 0.00 |
| S25 | 0.00 | 0.22 | 0.26 | 0.00 | 0.00 | 0.00 | 0.00 | 0.00 | 0.00 | 0.17 | 0.13 | 0.20 | 0.00 | 0.00 | 0.00 | 0.00 | 0.00 | 0.00 | 0.00 | 0.00 | 0.00 | 0.44 | 0.67 | 0.00 | 0.00 | 0.00 | 0.00 | 0.00 | 0.32 | 0.00 | 0.00 | 0.00 | 0.00 | 0.00 | 0.00 | 0.59 |
| S26 | 0.00 | 0.34 | 0.00 | 0.14 | 0.52 | 0.00 | 0.00 | 0.00 | 0.00 | 0.00 | 0.00 | 0.00 | -0.28 | 0.00 | 0.09 | 0.00 | 0.00 | 0.00 | 0.00 | 0.42 | 0.00 | 0.00 | 0.00 | 0.00 | 0.00 | 0.00 | 0.48 | 0.00 | 0.52 | 0.00 | 0.49 | 0.00 | 0.00 | 0.00 | 0.00 | 0.00 |
| S27 | 0.00 | 0.07 | 0.00 | 0.48 | -0.30 | 0.00 | 0.00 | 0.00 | 0.12 | 0.46 | 0.09 | 0.09 | 0.00 | 0.00 | 0.00 | 0.00 | 0.00 | 0.00 | 0.00 | 0.00 | 0.59 | 0.00 | 0.00 | 0.00 | 0.00 | 0.48 | 0.00 | 1.44 | 0.60 | 0.17 | 0.44 | 0.00 | 0.00 | 0.15 | 0.00 | 0.00 |
| S28 | 0.00 | 0.00 | 0.00 | 0.38 | -0.58 | 0.00 | 0.00 | 0.00 | 0.00 | 0.00 | 0.00 | -0.11 | -0.28 | 0.00 | 0.27 | 0.04 | 0.00 | 0.00 | 0.00 | 0.00 | 0.00 | 0.00 | 0.00 | 0.00 | 0.00 | 0.00 | 1.44 | 0.00 | 0.00 | 0.48 | 0.51 | 0.00 | -0.21 | 0.00 | 0.00 | 0.00 |
| S29 | 0.00 | 0.06 | 0.23 | 0.00 | 0.11 | 0.18 | 0.00 | 0.00 | 0.00 | 0.00 | 0.00 | 0.15 | 0.00 | 0.00 | 0.00 | 0.00 | 0.00 | 0.00 | 0.00 | 0.00 | 0.00 | 0.00 | 0.00 | 0.00 | 0.32 | 0.52 | 0.60 | 0.00 | 0.00 | 0.00 | 0.51 | -0.84 | 0.36 | 0.63 | 0.00 | 0.00 |
| S30 | 0.00 | 0.32 | 0.19 | 0.22 | -0.36 | 0.00 | 0.00 | 0.17 | 0.00 | 0.00 | 0.00 | 0.00 | 0.00 | 0.00 | 0.00 | 0.00 | 0.00 | 0.00 | 0.00 | 0.00 | 0.00 | 0.00 | 0.00 | 0.00 | 0.00 | 0.00 | 0.17 | 0.48 | 0.00 | 0.00 | 2.17 | 0.00 | 0.00 | 0.00 | 0.00 | 0.00 |
| S31 | 0.00 | 0.00 | 0.00 | 0.00 | 0.00 | 0.00 | 0.00 | 0.00 | 0.00 | 0.00 | 0.00 | 0.00 | 0.00 | 0.00 | 0.00 | 0.00 | 0.00 | 0.00 | 0.00 | 0.00 | 0.00 | 0.00 | 0.00 | 0.00 | 0.00 | 0.49 | 0.44 | 0.51 | 0.51 | 2.17 | 0.00 | 0.00 | 0.00 | 0.00 | 0.00 | 0.00 |
| S32 | 0.00 | -0.50 | 0.55 | 0.00 | 1.11 | 0.27 | 0.00 | 0.40 | 0.00 | 0.00 | 0.00 | 0.00 | -0.13 | 0.00 | -0.30 | 0.00 | 0.00 | 0.00 | 0.00 | 0.00 | 0.00 | 0.00 | 0.00 | 0.00 | 0.00 | 0.00 | 0.00 | 0.00 | -0.84 | 0.00 | 0.00 | 0.00 | 0.63 | 1.28 | 0.00 | 0.00 |
| S33 | 0.00 | -0.02 | 0.00 | -0.51 | 4.09 | 0.00 | 0.00 | 0.00 | 0.00 | -0.36 | 0.00 | 0.00 | 0.00 | 0.00 | -0.12 | 0.00 | 0.00 | 0.00 | 0.00 | 0.00 | 0.00 | 0.00 | 0.00 | 0.00 | 0.00 | 0.00 | 0.00 | -0.21 | 0.36 | 0.00 | 0.00 | 0.63 | 0.00 | -6.08 | 0.00 | 0.00 |
| S34 | 0.00 | 0.00 | 0.00 | 0.00 | 2.04 | 0.00 | 0.00 | 0.36 | 0.16 | 0.00 | 0.00 | 0.00 | 0.41 | 0.00 | 0.00 | 0.00 | 0.00 | 0.00 | 0.00 | 0.00 | 0.00 | 0.00 | 0.00 | 0.00 | 0.00 | 0.00 | 0.15 | 0.00 | 0.63 | 0.00 | 0.00 | 1.28 | -6.08 | 0.00 | 0.00 | 0.00 |
| S35 | 0.00 | 0.00 | 0.00 | 0.00 | 0.00 | 0.00 | 0.00 | 0.00 | 0.00 | 0.00 | 0.00 | 0.00 | -0.22 | 0.00 | 0.00 | 0.19 | 0.90 | 0.54 | 0.00 | 0.00 | 0.00 | 0.00 | 0.00 | 0.00 | 0.00 | 0.00 | 0.00 | 0.00 | 0.00 | 0.00 | 0.00 | 0.00 | 0.00 | 0.00 | 0.00 | 0.27 |
| S36 | 0.00 | 0.00 | 0.84 | 0.00 | 0.00 | 0.00 | 0.00 | 0.00 | 0.00 | 0.00 | 0.00 | 1.59 | 0.16 | 0.00 | 0.00 | 0.00 | 0.00 | 0.00 | 0.00 | 0.00 | 0.00 | 0.00 | 0.00 | 0.00 | 0.59 | 0.00 | 0.00 | 0.00 | 0.00 | 0.00 | 0.00 | 0.00 | 0.00 | 0.00 | 0.27 | 0.00 |

Table S2. Weight of each connection in the network of patients with 0-6m survival

Table S3. Weight of each connection in the network of patients with 6-12m survival

|  | S1 | S2 | S3 | S4 | S5 | S6 | S7 | S8 | S9 | S10 | S11 | S12 | S13 | S14 | S15 | S16 | S17 | S18 | S19 | S20 | S21 | S22 | S23 | S24 | S25 | S26 | S27 | S28 | S29 | S30 | S31 | S32 | S33 | S34 | S35 | S36 |
| --- | --- | --- | --- | --- | --- | --- | --- | --- | --- | --- | --- | --- | --- | --- | --- | --- | --- | --- | --- | --- | --- | --- | --- | --- | --- | --- | --- | --- | --- | --- | --- | --- | --- | --- | --- | --- |
| S1 | 0.00 | 0.00 | 0.00 | 0.00 | 0.54 | 0.00 | 0.00 | 0.00 | 0.00 | 0.00 | 0.00 | 0.00 | 0.00 | 0.00 | 0.00 | 0.00 | 0.00 | 0.00 | 0.00 | 0.00 | 0.00 | 0.00 | 0.00 | 0.00 | 0.00 | 0.00 | 0.00 | 0.00 | 0.00 | 0.00 | 0.00 | 0.00 | 0.00 | 0.00 | 0.00 | 0.00 |
| S2 | 0.00 | 0.00 | 0.00 | 0.00 | 0.00 | 0.00 | 0.00 | 0.00 | 0.00 | 0.00 | 0.00 | 0.00 | 0.00 | 0.00 | 0.00 | 0.00 | 0.00 | 0.00 | 0.00 | 0.00 | 0.00 | 0.00 | 0.00 | 0.00 | 0.00 | 0.00 | 0.00 | 0.00 | 0.00 | 0.00 | 0.00 | 0.00 | 0.00 | 0.00 | 0.00 | 0.00 |
| S3 | 0.00 | 0.00 | 0.00 | 0.00 | 0.00 | 0.00 | 0.00 | 0.00 | 0.00 | 0.00 | 0.00 | 0.00 | 0.00 | 0.00 | 0.00 | 0.00 | 0.00 | 0.00 | 0.00 | 0.00 | 0.00 | 0.00 | 0.00 | 0.00 | 0.00 | 0.00 | 0.00 | 0.00 | 0.00 | 0.00 | 0.00 | 0.00 | 0.00 | 0.00 | 0.00 | 0.00 |
| S4 | 0.00 | 0.00 | 0.00 | 0.00 | 0.00 | 0.00 | 0.87 | 0.00 | 0.00 | 0.00 | 0.00 | 0.00 | 0.00 | 0.00 | 0.00 | 0.00 | 0.00 | 0.00 | 0.00 | 0.76 | 0.00 | 0.00 | 0.00 | 0.00 | 0.00 | 0.00 | 0.00 | 0.00 | 0.00 | 0.00 | 0.00 | 0.00 | -0.73 | 0.00 | 0.00 | 0.00 |
| S5 | 0.54 | 0.00 | 0.00 | 0.00 | 0.00 | 0.00 | 0.00 | 0.00 | 0.00 | 0.00 | 0.00 | 0.00 | 0.00 | 0.00 | 0.00 | 0.00 | 0.00 | 0.00 | 0.00 | 0.00 | 0.00 | 0.00 | 0.00 | 0.00 | 0.00 | 0.00 | 0.00 | 0.00 | 0.00 | 0.00 | 0.00 | 0.00 | 2.50 | 0.00 | 0.00 | 0.00 |
| S6 | 0.00 | 0.00 | 0.00 | 0.00 | 0.00 | 0.00 | 1.61 | 0.00 | 0.00 | 0.00 | 0.00 | 0.00 | 0.00 | 0.00 | 0.00 | 0.00 | 0.00 | 0.00 | 0.00 | 0.00 | 0.00 | 0.00 | 0.00 | 0.00 | 0.00 | 0.00 | 0.00 | 0.00 | 0.00 | 0.00 | 0.00 | 0.00 | 0.00 | 0.00 | 0.00 | 0.00 |
| S7 | 0.00 | 0.00 | 0.00 | 0.87 | 0.00 | 1.61 | 0.00 | 0.00 | 0.00 | 0.00 | 0.00 | 0.00 | 0.00 | 0.00 | 0.00 | 0.00 | 0.00 | 0.00 | 0.00 | 0.00 | 0.00 | 0.00 | 0.00 | 0.00 | 0.00 | 0.00 | 0.00 | 0.00 | 0.00 | 0.00 | 0.00 | 0.00 | 0.00 | 0.00 | 0.00 | 0.00 |
| S8 | 0.00 | 0.00 | 0.00 | 0.00 | 0.00 | 0.00 | 0.00 | 0.00 | 0.00 | 0.00 | 0.00 | 0.00 | 0.00 | 0.00 | 0.00 | 0.00 | 0.00 | 0.00 | 0.00 | 0.00 | 0.00 | 0.00 | 0.00 | 0.00 | 0.00 | 0.00 | 0.00 | 0.00 | 0.00 | 0.00 | 0.00 | 0.00 | 0.00 | 0.00 | 0.00 | 0.00 |
| S9 | 0.00 | 0.00 | 0.00 | 0.00 | 0.00 | 0.00 | 0.00 | 0.00 | 0.00 | 2.54 | 0.00 | 0.00 | 0.00 | 0.00 | 0.00 | 0.00 | 0.00 | 0.00 | 0.00 | 0.00 | 0.00 | 0.00 | 0.00 | 0.00 | 0.00 | 0.00 | 0.00 | 0.00 | 0.00 | 0.00 | 0.00 | 0.00 | 0.00 | 0.00 | 0.00 | 0.00 |
| S10 | 0.00 | 0.00 | 0.00 | 0.00 | 0.00 | 0.00 | 0.00 | 0.00 | 2.54 | 0.00 | 0.00 | 0.00 | 0.00 | 0.00 | 0.00 | 0.00 | 0.00 | 0.00 | 0.00 | 0.00 | 0.00 | 0.00 | 0.00 | 0.00 | 0.00 | 0.00 | 0.00 | 0.00 | 0.00 | 0.00 | 0.00 | 0.00 | -0.13 | 0.00 | 0.00 | 0.00 |
| S11 | 0.00 | 0.00 | 0.00 | 0.00 | 0.00 | 0.00 | 0.00 | 0.00 | 0.00 | 0.00 | 0.00 | 0.00 | 0.00 | 0.00 | 0.00 | 0.00 | 0.00 | 0.00 | 0.00 | 0.00 | 0.00 | 0.00 | 0.00 | 0.00 | 0.00 | 0.00 | 0.00 | 0.00 | 0.00 | 0.00 | 0.00 | 0.00 | 0.00 | 0.00 | 0.00 | 0.00 |
| S12 | 0.00 | 0.00 | 0.00 | 0.00 | 0.00 | 0.00 | 0.00 | 0.00 | 0.00 | 0.00 | 0.00 | 0.00 | 1.29 | 0.00 | 0.00 | 0.00 | 0.00 | 0.00 | 0.00 | 0.00 | 0.00 | 0.00 | 0.00 | 0.00 | 0.00 | 0.00 | 0.00 | 0.00 | 0.00 | 0.00 | 0.00 | 0.00 | 0.00 | 0.00 | 0.00 | 1.20 |
| S13 | 0.00 | 0.00 | 0.00 | 0.00 | 0.00 | 0.00 | 0.00 | 0.00 | 0.00 | 0.00 | 0.00 | 1.29 | 0.00 | 0.00 | 0.00 | 0.00 | 0.00 | -0.35 | 0.00 | 0.00 | 0.00 | 0.00 | 0.00 | 0.00 | 0.00 | 0.00 | 0.00 | 0.00 | 0.00 | 0.00 | 0.00 | 0.00 | 0.00 | 0.00 | 0.00 | 0.00 |
| S14 | 0.00 | 0.00 | 0.00 | 0.00 | 0.00 | 0.00 | 0.00 | 0.00 | 0.00 | 0.00 | 0.00 | 0.00 | 0.00 | 0.00 | 0.00 | 0.00 | 0.00 | 0.00 | 0.00 | 0.00 | 0.00 | 0.00 | 0.00 | 0.00 | 0.00 | 0.00 | 0.00 | 0.00 | 0.00 | 0.00 | 0.00 | 0.00 | 0.00 | 0.00 | 0.00 | 0.00 |
| S15 | 0.00 | 0.00 | 0.00 | 0.00 | 0.00 | 0.00 | 0.00 | 0.00 | 0.00 | 0.00 | 0.00 | 0.00 | 0.00 | 0.00 | 0.00 | 3.02 | 0.15 | 1.21 | 0.00 | 0.00 | 0.00 | 0.00 | 0.00 | 0.00 | 0.00 | 0.00 | 0.00 | 0.00 | 0.00 | 0.00 | 0.00 | 0.00 | 0.00 | 0.00 | 0.00 | 0.00 |
| S16 | 0.00 | 0.00 | 0.00 | 0.00 | 0.00 | 0.00 | 0.00 | 0.00 | 0.00 | 0.00 | 0.00 | 0.00 | 0.00 | 0.00 | 3.02 | 0.00 | 0.00 | 0.00 | 0.87 | 0.00 | 0.00 | 0.00 | 0.00 | 0.00 | 0.00 | 0.00 | 0.00 | 0.00 | 0.00 | 0.00 | 0.00 | 0.00 | 0.00 | 0.00 | 0.00 | 0.00 |
| S17 | 0.00 | 0.00 | 0.00 | 0.00 | 0.00 | 0.00 | 0.00 | 0.00 | 0.00 | 0.00 | 0.00 | 0.00 | 0.00 | 0.00 | 0.15 | 0.00 | 0.00 | 0.00 | 0.00 | 0.86 | 0.00 | 0.00 | 0.00 | 0.00 | 0.00 | 0.00 | 0.00 | 0.00 | 0.00 | 0.00 | 0.00 | 0.00 | 0.00 | 0.00 | 0.00 | 0.00 |
| S18 | 0.00 | 0.00 | 0.00 | 0.00 | 0.00 | 0.00 | 0.00 | 0.00 | 0.00 | 0.00 | 0.00 | 0.00 | -0.35 | 0.00 | 1.21 | 0.00 | 0.00 | 0.00 | 0.00 | 1.35 | 0.00 | 0.00 | 0.00 | 0.00 | 0.00 | 0.00 | 0.00 | 0.00 | 0.00 | 0.00 | 0.00 | 0.00 | 0.00 | 0.00 | 1.05 | 0.00 |
| S19 | 0.00 | 0.00 | 0.00 | 0.00 | 0.00 | 0.00 | 0.00 | 0.00 | 0.00 | 0.00 | 0.00 | 0.00 | 0.00 | 0.00 | 0.00 | 0.87 | 0.00 | 0.00 | 0.00 | 0.00 | 0.00 | 0.00 | 0.00 | 0.00 | 0.00 | 0.00 | 0.00 | 0.00 | 0.00 | 0.00 | 0.00 | 0.00 | 0.00 | 0.00 | 0.00 | 0.00 |
| S20 | 0.00 | 0.00 | 0.00 | 0.76 | 0.00 | 0.00 | 0.00 | 0.00 | 0.00 | 0.00 | 0.00 | 0.00 | 0.00 | 0.00 | 0.00 | 0.00 | 0.86 | 1.35 | 0.00 | 0.00 | 0.00 | 0.00 | 0.00 | 0.00 | 0.00 | 0.00 | 0.00 | 0.00 | 0.00 | 0.00 | 0.00 | 0.00 | 0.00 | 0.00 | 0.00 | 0.00 |
| S21 | 0.00 | 0.00 | 0.00 | 0.00 | 0.00 | 0.00 | 0.00 | 0.00 | 0.00 | 0.00 | 0.00 | 0.00 | 0.00 | 0.00 | 0.00 | 0.00 | 0.00 | 0.00 | 0.00 | 0.00 | 0.00 | 2.57 | 0.00 | 0.00 | 0.00 | 0.00 | 0.00 | 0.00 | 0.00 | 0.00 | 0.00 | 0.00 | 0.00 | 0.00 | 0.00 | 0.00 |
| S22 | 0.00 | 0.00 | 0.00 | 0.00 | 0.00 | 0.00 | 0.00 | 0.00 | 0.00 | 0.00 | 0.00 | 0.00 | 0.00 | 0.00 | 0.00 | 0.00 | 0.00 | 0.00 | 0.00 | 0.00 | 2.57 | 0.00 | 0.00 | 0.00 | 0.00 | 0.00 | 0.00 | 0.00 | 0.00 | 0.00 | 0.00 | 0.00 | 0.00 | 0.00 | 0.00 | 0.00 |
| S23 | 0.00 | 0.00 | 0.00 | 0.00 | 0.00 | 0.00 | 0.00 | 0.00 | 0.00 | 0.00 | 0.00 | 0.00 | 0.00 | 0.00 | 0.00 | 0.00 | 0.00 | 0.00 | 0.00 | 0.00 | 0.00 | 0.00 | 0.00 | 0.00 | 0.00 | 0.00 | 0.00 | 0.00 | 0.00 | 0.00 | 0.00 | 0.00 | 0.00 | 0.00 | 0.00 | 0.00 |
| S24 | 0.00 | 0.00 | 0.00 | 0.00 | 0.00 | 0.00 | 0.00 | 0.00 | 0.00 | 0.00 | 0.00 | 0.00 | 0.00 | 0.00 | 0.00 | 0.00 | 0.00 | 0.00 | 0.00 | 0.00 | 0.00 | 0.00 | 0.00 | 0.00 | 0.00 | 0.00 | 0.00 | 0.00 | 0.00 | 0.00 | 0.00 | 0.00 | 0.00 | 0.00 | 0.00 | 0.00 |
| S25 | 0.00 | 0.00 | 0.00 | 0.00 | 0.00 | 0.00 | 0.00 | 0.00 | 0.00 | 0.00 | 0.00 | 0.00 | 0.00 | 0.00 | 0.00 | 0.00 | 0.00 | 0.00 | 0.00 | 0.00 | 0.00 | 0.00 | 0.00 | 0.00 | 0.00 | 0.00 | 0.00 | 0.00 | 0.00 | 0.00 | 0.00 | 0.00 | 0.00 | 0.00 | 0.00 | 0.00 |
| S26 | 0.00 | 0.00 | 0.00 | 0.00 | 0.00 | 0.00 | 0.00 | 0.00 | 0.00 | 0.00 | 0.00 | 0.00 | 0.00 | 0.00 | 0.00 | 0.00 | 0.00 | 0.00 | 0.00 | 0.00 | 0.00 | 0.00 | 0.00 | 0.00 | 0.00 | 0.00 | 0.00 | 0.00 | 0.36 | 0.00 | 0.00 | 0.00 | 0.00 | 0.00 | 0.00 | 0.00 |
| S27 | 0.00 | 0.00 | 0.00 | 0.00 | 0.00 | 0.00 | 0.00 | 0.00 | 0.00 | 0.00 | 0.00 | 0.00 | 0.00 | 0.00 | 0.00 | 0.00 | 0.00 | 0.00 | 0.00 | 0.00 | 0.00 | 0.00 | 0.00 | 0.00 | 0.00 | 0.00 | 0.00 | 0.00 | 0.00 | 0.00 | 0.00 | 0.00 | -0.54 | 0.00 | 0.00 | 0.00 |
| S28 | 0.00 | 0.00 | 0.00 | 0.00 | 0.00 | 0.00 | 0.00 | 0.00 | 0.00 | 0.00 | 0.00 | 0.00 | 0.00 | 0.00 | 0.00 | 0.00 | 0.00 | 0.00 | 0.00 | 0.00 | 0.00 | 0.00 | 0.00 | 0.00 | 0.00 | 0.00 | 0.00 | 0.00 | 0.00 | 0.00 | 0.00 | 0.00 | 0.00 | 0.00 | 0.00 | 0.00 |
| S29 | 0.00 | 0.00 | 0.00 | 0.00 | 0.00 | 0.00 | 0.00 | 0.00 | 0.00 | 0.00 | 0.00 | 0.00 | 0.00 | 0.00 | 0.00 | 0.00 | 0.00 | 0.00 | 0.00 | 0.00 | 0.00 | 0.00 | 0.00 | 0.00 | 0.00 | 0.36 | 0.00 | 0.00 | 0.00 | 0.00 | 0.00 | 0.00 | 0.00 | 0.00 | 0.00 | 0.00 |
| S30 | 0.00 | 0.00 | 0.00 | 0.00 | 0.00 | 0.00 | 0.00 | 0.00 | 0.00 | 0.00 | 0.00 | 0.00 | 0.00 | 0.00 | 0.00 | 0.00 | 0.00 | 0.00 | 0.00 | 0.00 | 0.00 | 0.00 | 0.00 | 0.00 | 0.00 | 0.00 | 0.00 | 0.00 | 0.00 | 0.00 | 1.97 | 0.00 | 0.00 | 0.00 | 0.00 | 0.00 |
| S31 | 0.00 | 0.00 | 0.00 | 0.00 | 0.00 | 0.00 | 0.00 | 0.00 | 0.00 | 0.00 | 0.00 | 0.00 | 0.00 | 0.00 | 0.00 | 0.00 | 0.00 | 0.00 | 0.00 | 0.00 | 0.00 | 0.00 | 0.00 | 0.00 | 0.00 | 0.00 | 0.00 | 0.00 | 0.00 | 1.97 | 0.00 | 0.00 | 0.00 | 0.00 | 0.00 | 0.00 |
| S32 | 0.00 | 0.00 | 0.00 | 0.00 | 0.00 | 0.00 | 0.00 | 0.00 | 0.00 | 0.00 | 0.00 | 0.00 | 0.00 | 0.00 | 0.00 | 0.00 | 0.00 | 0.00 | 0.00 | 0.00 | 0.00 | 0.00 | 0.00 | 0.00 | 0.00 | 0.00 | 0.00 | 0.00 | 0.00 | 0.00 | 0.00 | 0.00 | 0.00 | 0.00 | 0.00 | 0.00 |
| S33 | 0.00 | 0.00 | 0.00 | -0.73 | 2.50 | 0.00 | 0.00 | 0.00 | 0.00 | -0.13 | 0.00 | 0.00 | 0.00 | 0.00 | 0.00 | 0.00 | 0.00 | 0.00 | 0.00 | 0.00 | 0.00 | 0.00 | 0.00 | 0.00 | 0.00 | 0.00 | -0.54 | 0.00 | 0.00 | 0.00 | 0.00 | 0.00 | 0.00 | -1.93 | 0.00 | 0.00 |
| S34 | 0.00 | 0.00 | 0.00 | 0.00 | 0.00 | 0.00 | 0.00 | 0.00 | 0.00 | 0.00 | 0.00 | 0.00 | 0.00 | 0.00 | 0.00 | 0.00 | 0.00 | 0.00 | 0.00 | 0.00 | 0.00 | 0.00 | 0.00 | 0.00 | 0.00 | 0.00 | 0.00 | 0.00 | 0.00 | 0.00 | 0.00 | 0.00 | -1.93 | 0.00 | 0.00 | 0.00 |
| S35 | 0.00 | 0.00 | 0.00 | 0.00 | 0.00 | 0.00 | 0.00 | 0.00 | 0.00 | 0.00 | 0.00 | 0.00 | 0.00 | 0.00 | 0.00 | 0.00 | 0.00 | 1.05 | 0.00 | 0.00 | 0.00 | 0.00 | 0.00 | 0.00 | 0.00 | 0.00 | 0.00 | 0.00 | 0.00 | 0.00 | 0.00 | 0.00 | 0.00 | 0.00 | 0.00 | 0.00 |
| S36 | 0.00 | 0.00 | 0.00 | 0.00 | 0.00 | 0.00 | 0.00 | 0.00 | 0.00 | 0.00 | 0.00 | 1.20 | 0.00 | 0.00 | 0.00 | 0.00 | 0.00 | 0.00 | 0.00 | 0.00 | 0.00 | 0.00 | 0.00 | 0.00 | 0.00 | 0.00 | 0.00 | 0.00 | 0.00 | 0.00 | 0.00 | 0.00 | 0.00 | 0.00 | 0.00 | 0.00 |

Table S4. Node strength in the network of patients with 0-6m survival

| Node | Strength |
| --- | --- |
| S1 | -0.91 |
| S2 | -0.20 |
| S3 | -0.50 |
| S4 | 0.13 |
| S5 | 4.03 |
| S6 | 0.14 |
| S7 | 0.02 |
| S8 | -0.82 |
| S9 | 0.03 |
| S10 | 0.52 |
| S11 | -1.13 |
| S12 | 0.30 |
| S13 | 0.57 |
| S14 | -1.12 |
| S15 | 0.95 |
| S16 | 0.16 |
| S17 | 0.37 |
| S18 | 0.12 |
| S19 | -0.46 |
| S20 | 0.08 |
| S21 | -0.31 |
| S22 | -0.22 |
| S23 | -1.00 |
| S24 | -1.46 |
| S25 | -0.60 |
| S26 | -0.52 |
| S27 | 0.10 |
| S28 | -0.23 |
| S29 | -0.17 |
| S30 | -0.29 |
| S31 | -0.28 |
| S32 | 0.25 |
| S33 | 2.07 |
| S34 | 1.71 |
| S35 | -0.85 |

Table S5. Node strength in the network of patients with 6-12m survival

| Node | Strength |
| --- | --- |
| S1 | -0.69 |
| S2 | -1.05 |
| S3 | -1.05 |
| S4 | 0.52 |
| S5 | 0.97 |
| S6 | 0.02 |
| S7 | 0.60 |
| S8 | -1.05 |
| S9 | 0.64 |
| S10 | 0.73 |
| S11 | -1.05 |
| S12 | 0.61 |
| S13 | 0.04 |
| S14 | -1.05 |
| S15 | 1.86 |
| S16 | 1.54 |
| S17 | -0.38 |
| S18 | 1.58 |
| S19 | -0.47 |
| S20 | 0.92 |
| S21 | 0.66 |
| S22 | 0.66 |
| S23 | -1.05 |
| S24 | -1.05 |
| S25 | -1.05 |
| S26 | -0.81 |
| S27 | -0.69 |
| S28 | -1.05 |
| S29 | -0.81 |
| S30 | 0.26 |
| S31 | 0.26 |
| S32 | -1.05 |
| S33 | 2.83 |
| S34 | 0.24 |
| S35 | -0.35 |
| S36 | -0.25 |


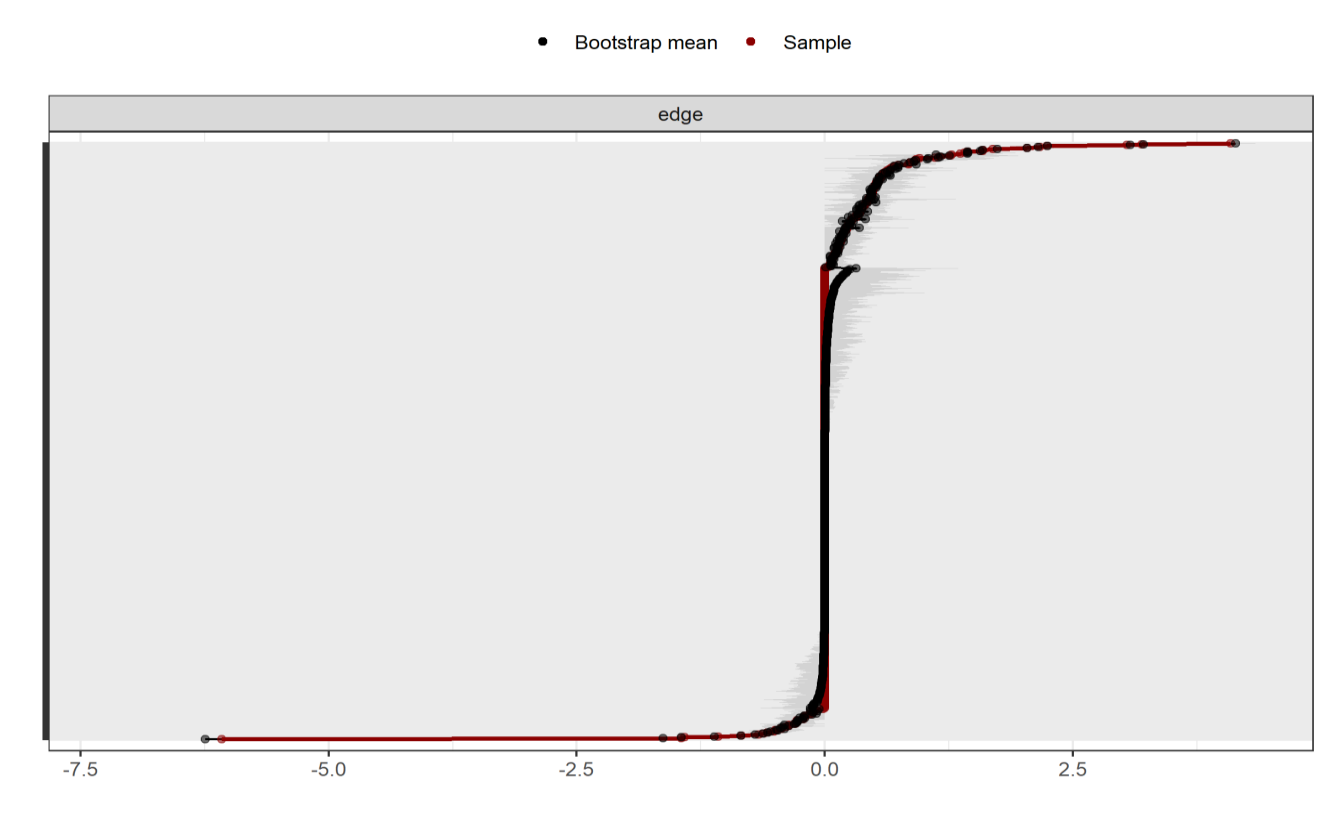

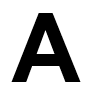

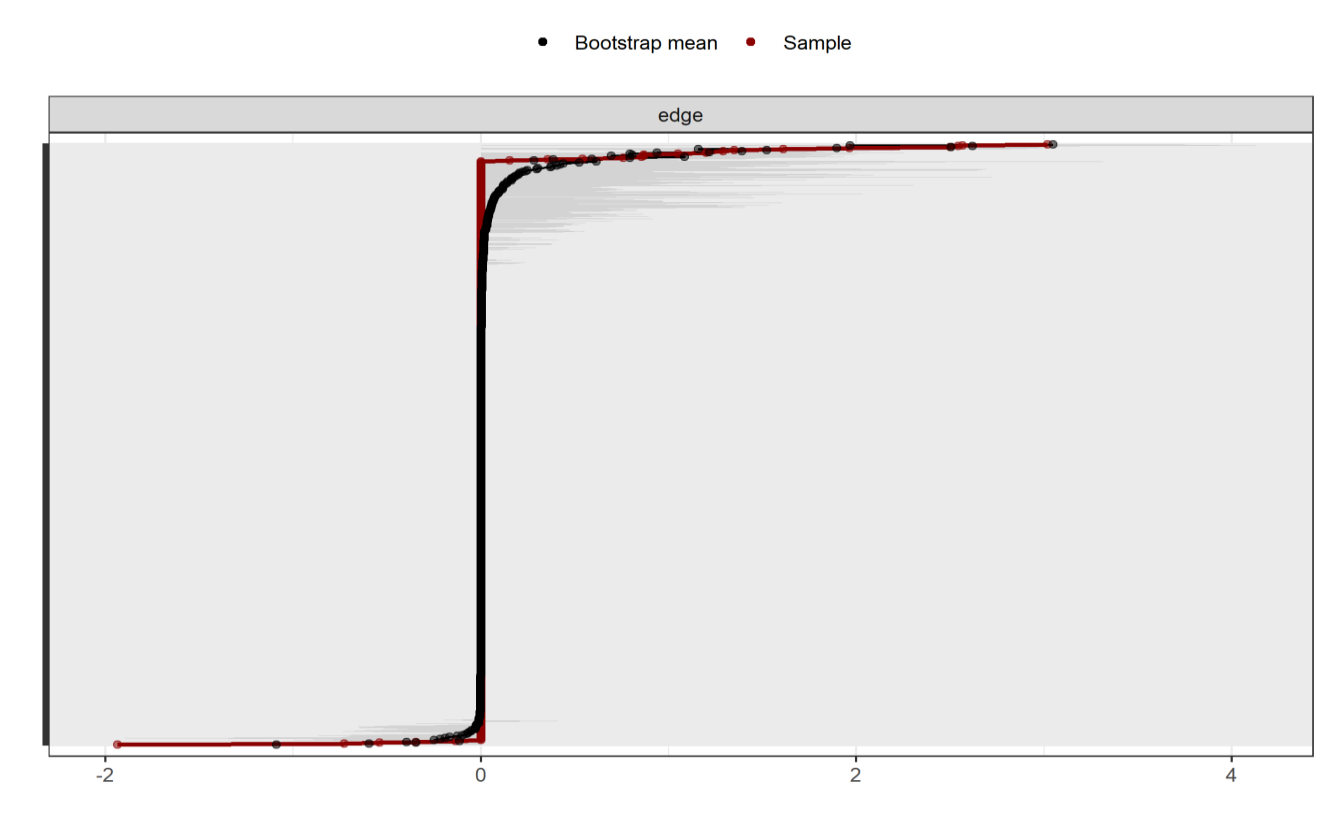

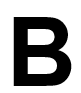


Figure S1. Bootstrapped confidence intervals of edge weights

Footnote: (A) Bootstrapped confidence intervals of edge weights in the network of patients with 0-6m survival; (B) Bootstrapped confidence intervals of edge weights in the network of patients with 6–12 m survival. Edges ordered from strongest (top) to weakest (bottom). Points indicate the mean edge weights and lines indicates the 95% CI. Node labels removed for legibility.


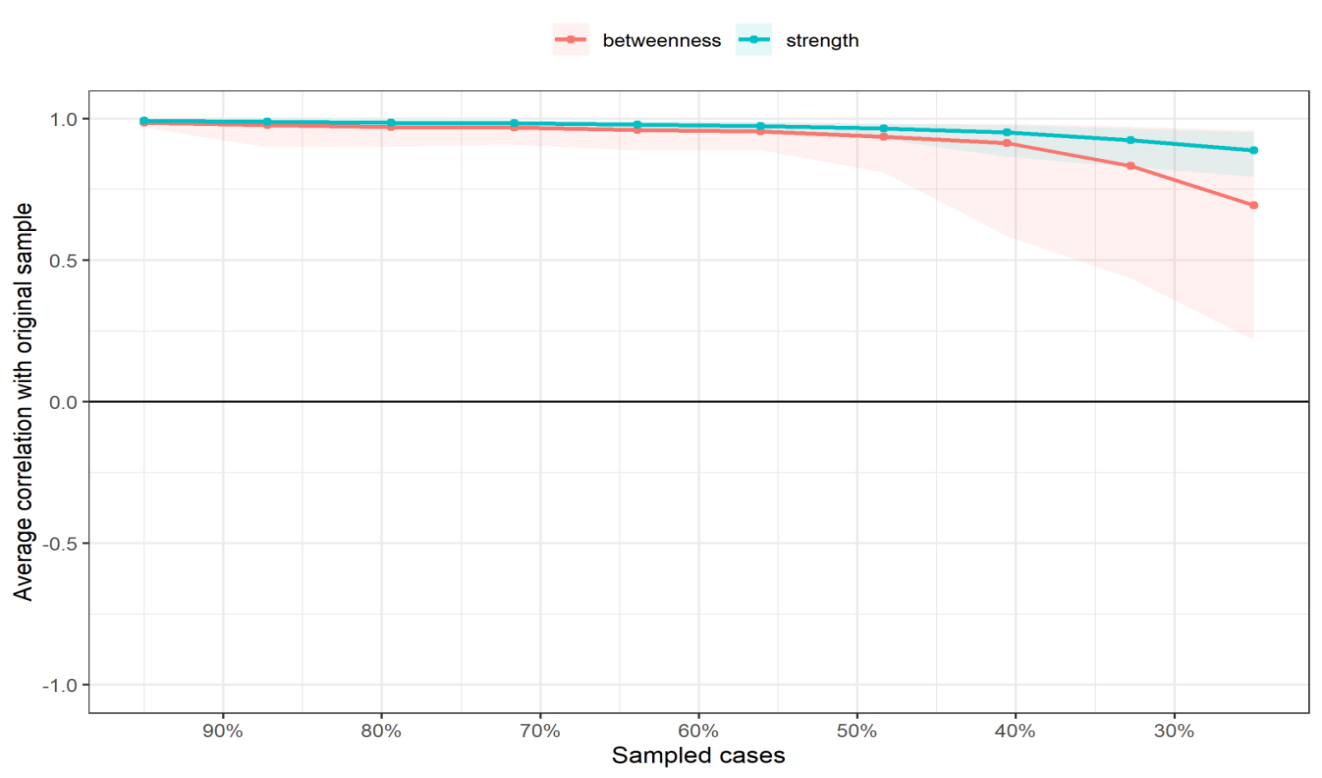

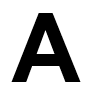

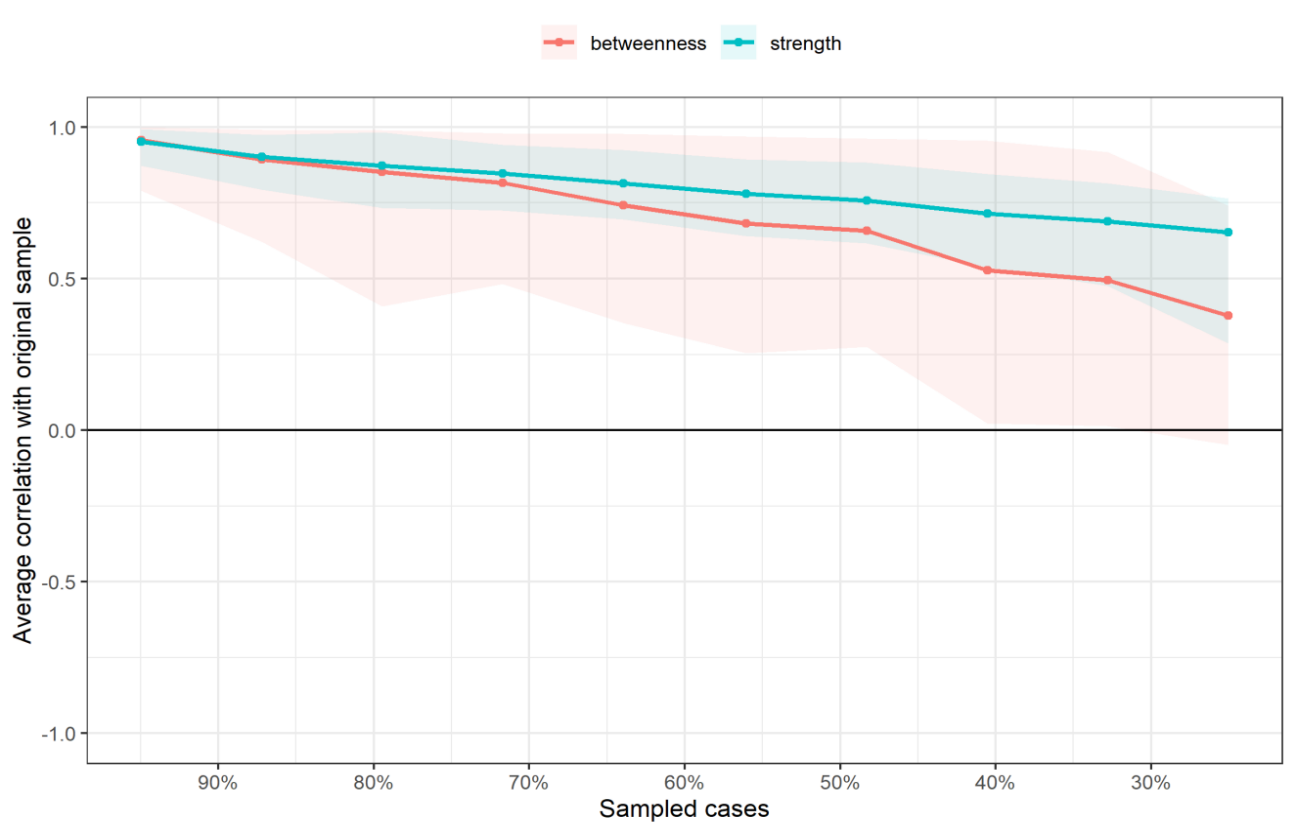

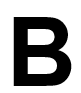


Figure S2. Stability of the centrality indices

Footnote: (A) Stability of the centrality indices in the network of patients with 0-6m survival; (B) Stability of the centrality indices in the network of patients with 6–12 m survival. Lines represent the mean correlations between the subsamples and the total sample; shaded areas represent the 95% CI of the sampled estimates. The statistic closeness does not contain any variance and is therefore not shown.


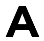

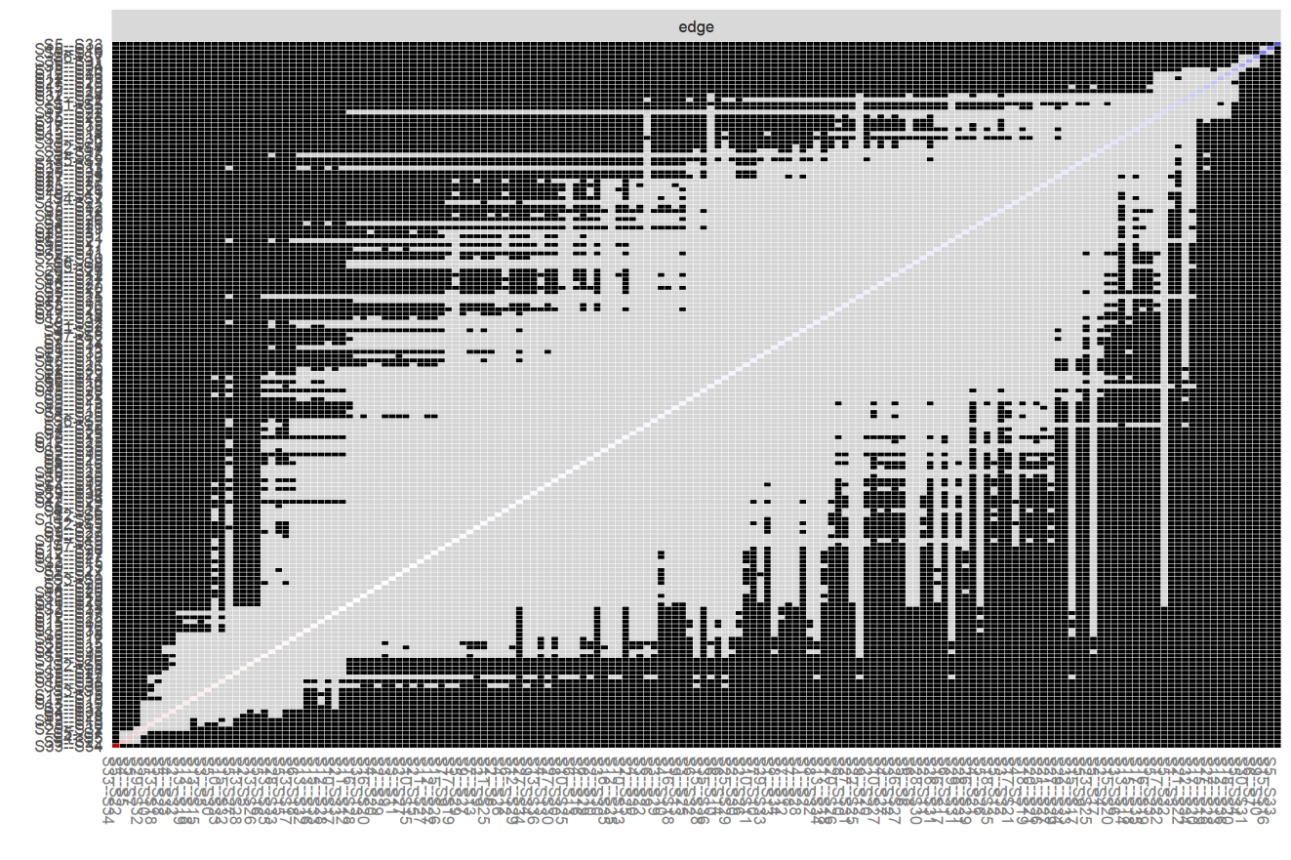

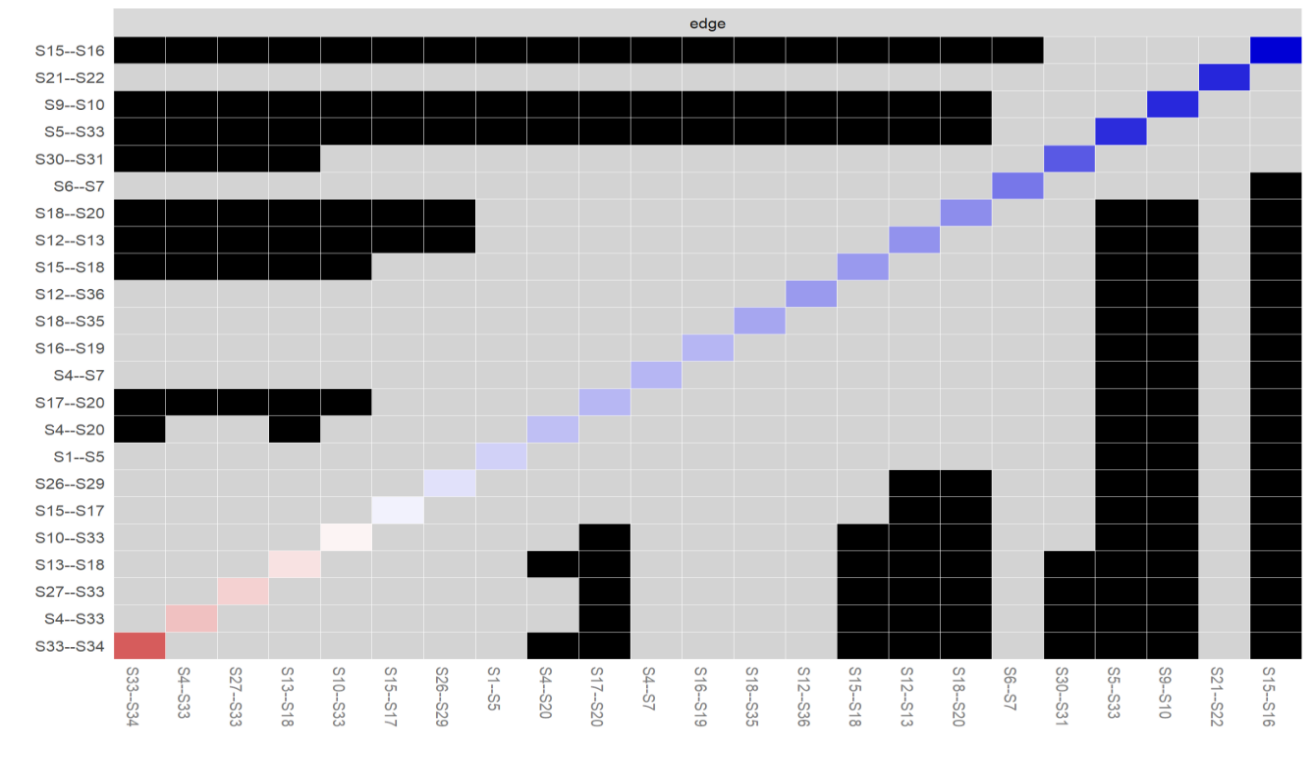

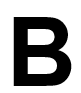


Figure S3. Estimation of edge weight difference

Footnote: (A) Estimation of edge weight difference in the network of patients with 0-6m survival; Estimation of edge weight difference in the network of patients with 6–12m survival. Matrix comparing all significant edge weights (0–6 month). Gray boxes indicate edges that do not significantly differ from one-another. Black boxes represent edges with significant difference from one another (α = 0.05). Blue boxes in the edge-weight plot indicate positive correlations.


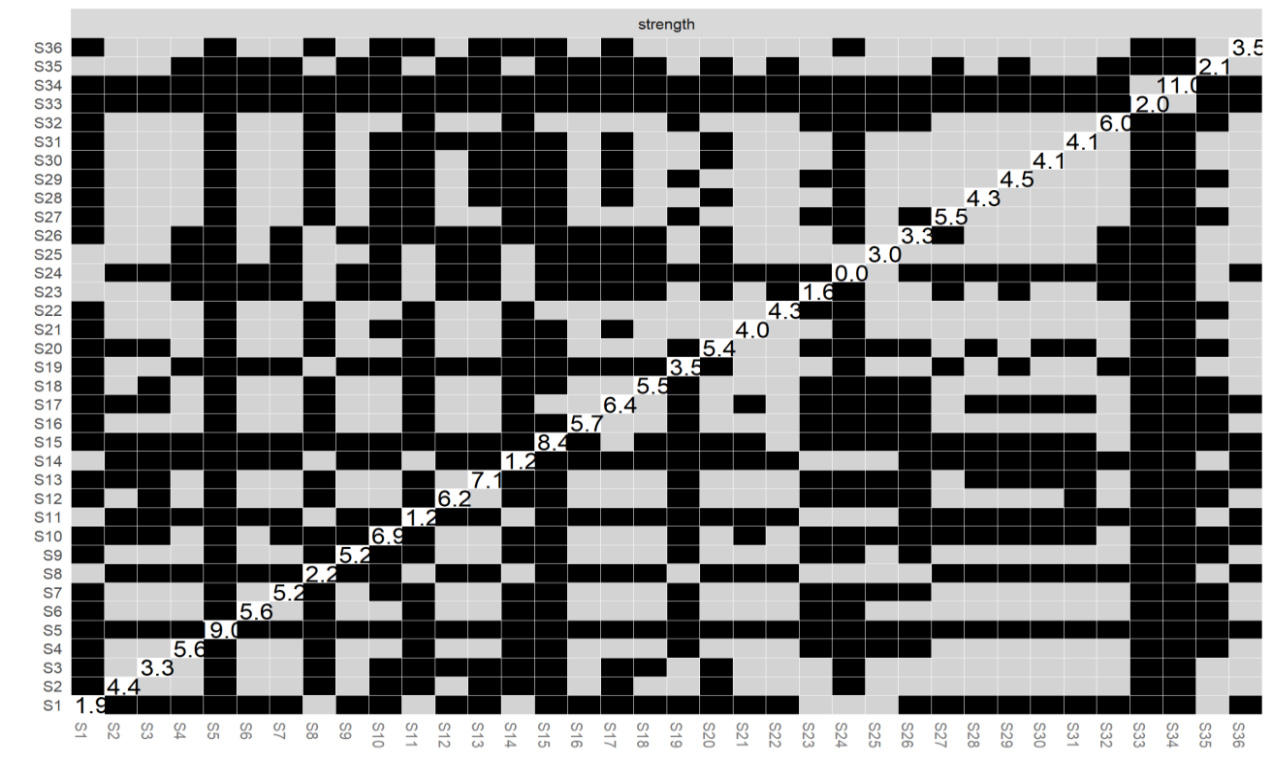

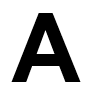

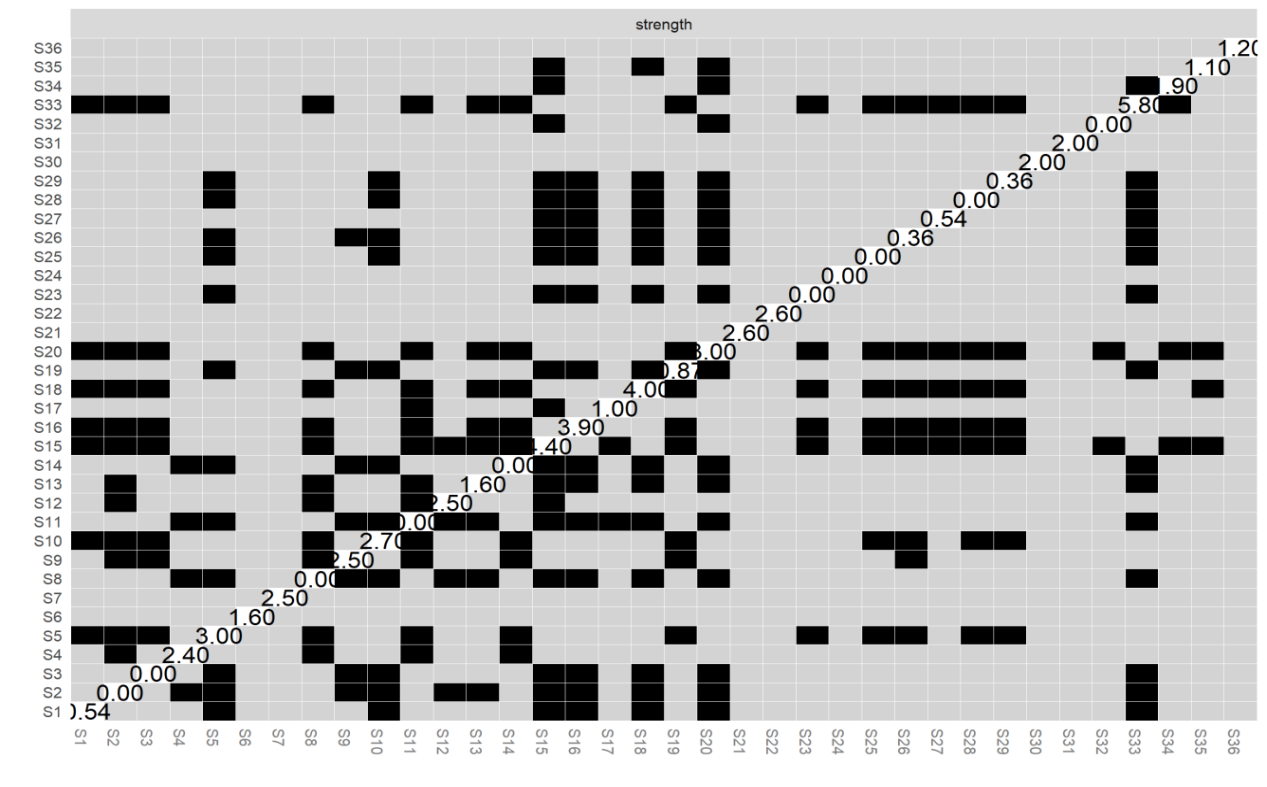

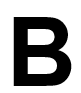


Figure S4. Estimation of node strength difference

Footnote: (A) Node strength difference test in the network of patients with 0-6m survival; (B) Node strength difference test in the network of patients with 6–12 m survival. Gray boxes indicate nodes that do not significantly differ from one-another. Black boxes represent nodes that differ significantly from one another (α = 0.05). White boxes show the values of node strength.
